# Tissue-specific DNA isolation from dissected millipedes for nanopore sequencing

**DOI:** 10.1093/biomethods/bpaf042

**Published:** 2025-05-28

**Authors:** Elena Cruz, William Wittstock, Bruce A Snyder, Arnab Sengupta

**Affiliations:** Department of Biological and Environmental Sciences, Georgia College & State University, Milledgeville, GA 31061, United States; Department of Biological and Environmental Sciences, Georgia College & State University, Milledgeville, GA 31061, United States; Department of Biological and Environmental Sciences, Georgia College & State University, Milledgeville, GA 31061, United States; Department of Biological and Environmental Sciences, Georgia College & State University, Milledgeville, GA 31061, United States

**Keywords:** millipede, DNA, long-read sequencing, nanopore, mitochondrial DNA

## Abstract

There are approximately 12,000 described species within the class Diplopoda. Only five species, falling within 4 of 16 described orders, have fully sequenced genomes. No whole genomes are available for incredibly diverse families like Xystodesmidae. Furthermore, genetic information attributed to key functions in these species is very limited. There is a growing interest in characterizing genomes of non-model organisms, however, extracting high-quality DNA for organisms with complex morphology can be challenging. Here we describe a detailed methodology for obtaining high-purity DNA from legs, head, and body tissues from wild-caught specimens of the millipede species *Cherokia georgiana*. Our dissection protocol separates the digestive tract minimizing microbial abundance in the extracted DNA sample. We describe sample homogenization steps that improve total DNA yield. To assess sample quality, concentration, and size we use spectrophotometry, fluorometry, and automated electrophoresis, respectively. We consistently obtain average DNA length upwards of 12–25 kb. We applied Oxford Nanopore Technologies MinION long-read sequencing, an affordable and accessible option with potential for field-based applications. Here we present tissue-specific DNA sequencing metrics, alignment and assembly of mitochondrial DNA consensus sequence, and phylogenetic analysis. While noting the limitations of our nanopore-based sequencing methodology, we provide a framework to process field specimens for PCR-free DNA sequencing data that can be used for gene-specific alignment and analysis.

## Introduction

Millipedes are some of the oldest terrestrial animals in the fossil record and their lineage dates back over 400 million years [1, 2]. These arthropods fulfill vital ecosystem roles such as maintaining soil structure, nutrient cycling, and serving as a crucial part of the food web [3–5]. The diversity of millipedes across habitats worldwide reflects their enduring nature and adaptability, with thousands of species thriving in different ecosystems [4].

Genetic analysis will enable molecular and biochemical characterization of critical aspects of millipede adaptations to diverse habitats, reproductive strategies, and interactions with other organisms [6]. Such studies are essential for addressing existing gaps in our understanding of millipede diversity and evolution [7]. Despite their evolutionary and ecological significance and the advancements in genomic sequencing technology, only five of the >12,000 described species of millipedes have a published full genome assembly [4, 8]. Among these species we observe a relatively wide diversity of genome sizes, and, interestingly, broad variability in horizontal gene transfer events across sequenced millipedes [8]. Furthermore, whole genome sequencing presents opportunities for discovery of new genes and regulatory features from understudied non-model organisms [9].

Next-generation sequencing (NGS) technologies offer several applications that aide in describing the genetics and evolution of an uncharacterized organism, such as phylogenetic and evolutionary analysis (mtDNA, ribosomal RNA), novel gene discovery (whole genome or transcriptome *de novo* assembly), and functional and comparative genomic analysis (RNA-seq). All NGS technologies require high sample purity in addition to high DNA concentration, and third-generation sequencing technologies further require high molecular weight (HMW) genomic DNA [10]. Arthropods pose unique challenges for extracting high-quality HMW DNA due to complex morphological and anatomical features, such as a tough exoskeleton [11]. Sclerotization of the exoskeleton in millipedes often incorporates calcium carbonate for rigidity and durability [12]. The robust exoskeleton impedes efficient cell lysis during extraction protocols, necessitating vigorous homogenization procedures, often resulting in fragmented DNA [13]. Unlike some arthropods, millipedes lack a pupal stage that typically offers easier access to soft tissues which are best suited for DNA extraction [14]. Millipede DNA extraction is challenging due to recalcitrant exoskeleton components, including chitin [15].

Moreover, millipedes host diverse microbial communities within their digestive tract, leading to additional complexities in sequencing data analysis [16]. While sequencing a whole millipede specimen is feasible, including the gut microbiome can be bioinformatically redundant [17, 18]. High levels of microbial DNA also increase the likelihood of mischaracterizing the specimen DNA [19]. Finally, certain specialized applications like Hi-C sequencing for genome-wide chromatin interactions require soft-tissue samples in order to preserve chromatin structure [20].

**Figure 1.**
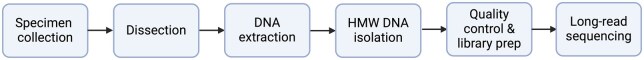
Workflow depicting specimen processing and long-read sequencing. Overview of HMW DNA extraction and subsequent quality assurance methods used in this study. Created using BioRender.

Here we present the detailed methodology (Fig. 1) for millipede specimen dissection into three distinct tissue types—legs, head, and body. The goal in benchmarking DNA extraction from each tissue type is to enable users to select DNA tissue sources based on specific applications. We delineate advantages and limitations of each tissue type. Our dissection strategy separates the intact digestive tract from other tissues, enabling targeted analysis of the gut microbiome. We consider metrics including DNA concentration, total yield, DNA size, and purity. We developed this protocol on the millipede *Cherokia georgiana* [21], a member of the most diverse millipede order (Polydesmida) and one of the most diverse millipede families (Xystodesmidae). The taxonomy of *C. georgiana* has recently been clarified and may be a good candidate for future genomic and transcriptomic studies because of its variability in coloration and body proportions across its range in the southeastern United States [22].

Furthermore, we apply ONT long-read sequencing on tissue-specific DNA. While we recognize that ONT sequencing on the MinION platform has limited utility due to accuracy and depth, it offers certain clear advantages. Specimen DNA can be sequenced PCR-free and the portability of the device enables data collection at field sites. Furthermore, the analysis pipeline with EPI2ME workflows for reference-guided alignment is easy-to-use and robust. We align sequencing data from each tissue type to a related reference mitogenome, assembly mtDNA from each tissue, compare assemblies for variants, and obtain a consensus sequence. Finally, we map the consensus mtDNA with annotations and build a phylogenetic tree. Our work highlights the utility of nanopore sequencing in tissue-specific DNA analysis in millipedes.

## Materials and methods

### Sample collection

Live adult male *C. georgiana* specimens were hand collected from two locations in Baldwin County, Georgia, USA for genomic DNA extraction. Our primary collection site was Lake Laurel Biological Field Station on Georgia College & State University’s East Campus (33.118°N, 83.184°W). Some specimens were also collected from a residential backyard located near the city center. Millipedes were kept alive until DNA extraction in plastic bins filled with soil and leaf litter from the collection site as both a habitat and food source. Specimen containers were sprayed with tap water regularly (1–2 times a week). Only mature adult males were selected for genomic DNA extraction; these were confirmed as *C. georgiana* based on mature gonopod anatomy using a dissecting microscope (Labomed Luxeo 6Z). Prior to dissection and DNA extraction, specimens were euthanized by submerging in 95% ethanol for ∼10 min.

### Dissection and tissue preparation

**Figure 2.**
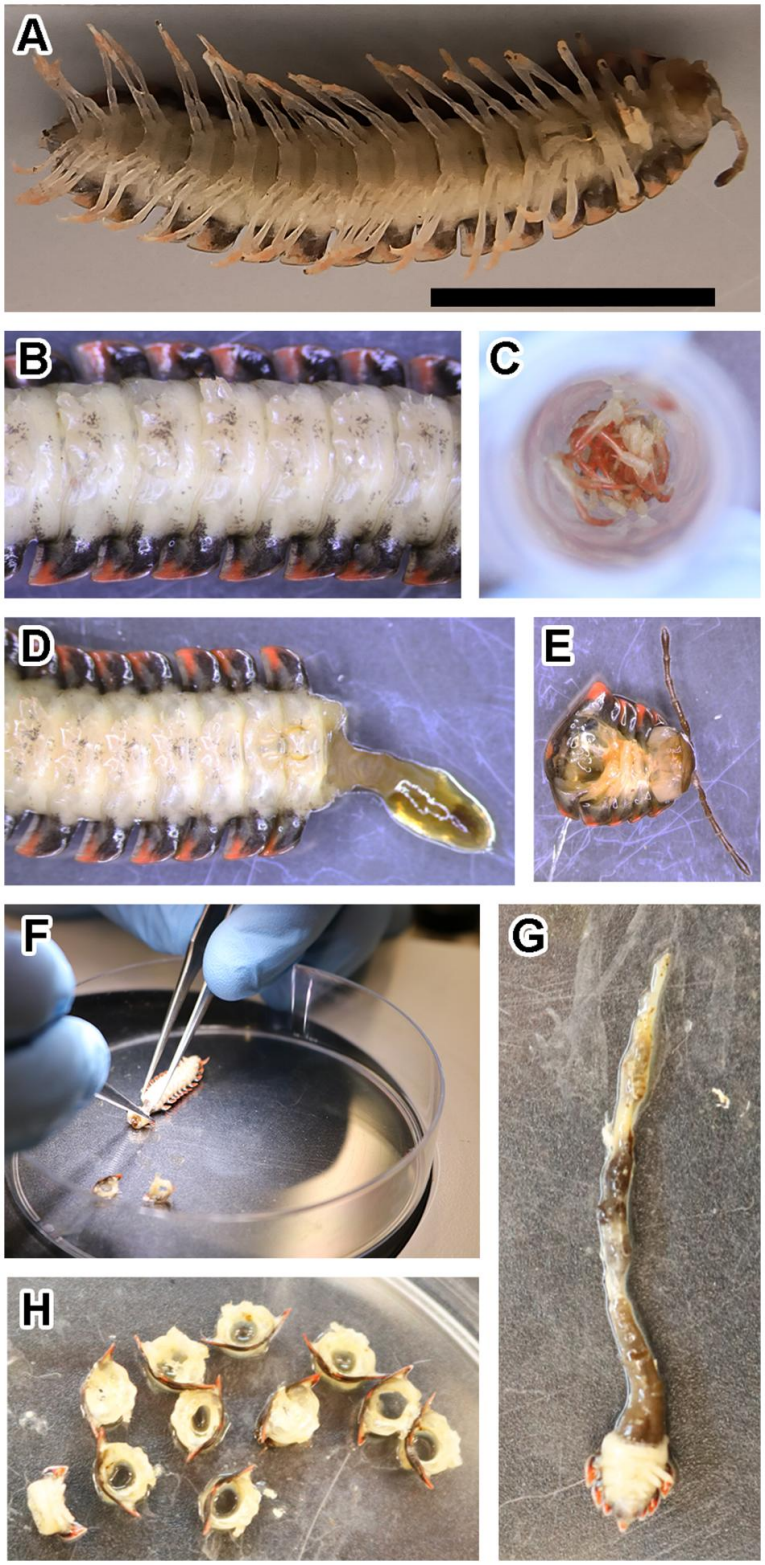
*Cherokia georgiana* dissection. Body with legs removed (A). Legs placed into 1.5 ml microcentrifuge tube (B). Head removed by incision between segments 4 and 5 (C and D). Body rings individually removed from specimen (E). The digestive tract with last three body segments still attached (F). Body rings and attached soft tissue used in DNA extraction (G)

**Figure 3.**
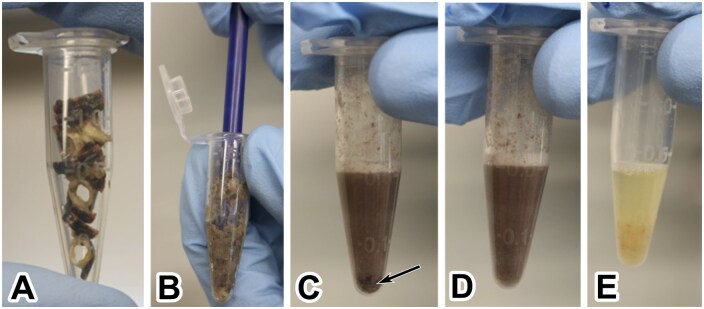
Homogenization of dissected tissue. After dissection, the body segments (A), head, and legs are placed in separate 1.5 ml microcentrifuge tubes and are sufficiently crushed using a pestle. The pestle is pressed against the tissue and the wall of each microcentrifuge tube to further homogenize the sample until the crunching of exoskeleton was no longer audible, typically around 20 min (B). The body segments (C) typically needed more time crushing than the head (D) or leg (E) tissue. Arrow indicates compacted material that needed to be resuspended and further homogenized

Deceased specimens were removed from ethanol and placed in a petri dish under a dissecting microscope (Labomed Luxeo 6Z). Single whole millipedes were used for analysis, with each individual isolated into three separate tissue types. Sterilized fine-point forceps were used to remove each leg at its base (near the sternite) and placed in a 1.5 ml microcentrifuge tube (Fig. 2A–B). Sample tubes were left uncapped to allow excess ethanol to evaporate prior to DNA extraction. Following the removal of the legs, the body, and head tissues were separated between the fourth and fifth leg-bearing segment (Fig. 2C–D). The head portion containing both the head and the first five segments was then stored in a separate tube. To prevent puncturing of the gut, the remaining body segments were removed individually (Figs. 2E–G, 3A). Segments were separated using forceps such that the digestive tract remained intact and unpunctured (Fig. 2F). A blunt pair of forceps held the specimen in place, while fine-point forceps were used to sever the tissue between segments and slide the body ring off of the intact digestive tract (Fig. 2E). The digestive tract was left attached to the last three segments to limit its contact with other soft tissues. These parts were stored in 95% ethanol with the specimen gonopods.

Respective tissue types were weighed on a microscale. Excess ethanol was removed by pipetting or evaporation. DNA extraction was carried out immediately after dissection to prevent tissue degradation.

### Extraction of high-purity DNA

Omega Biotek E.Z.N.A. Insect DNA Kit (Cat. No. D0926-01) was used for genomic DNA extraction. The manufacturer’s protocol was adjusted to maximize yield. Extractions on the three tissue types were carried out separately in six replicate specimens, with no sample pooling. Filtered sterile pipette tips and nuclease-free water were used for DNA extraction and quantitation. Kit contents were prepared per manufacturer’s instructions. We also note that we tested a few other methods for DNA extraction, however, these resulted in low quality DNA extracts from our millipede species (Supplementary Fig. S1).

Using a sterilized pestle (RPI Corp., Cat No. 12-141-366), body segments, head, and legs were thoroughly crushed until pieces of tissue were visibly homogenous and fluid in consistency (Fig. 3B). Exceeding the recommended tissue weight for the kit resulted in a higher DNA yield, potentially attributable to the additional soft tissue attached to the exoskeleton. Prior to the addition of CTL buffer (cell lysis buffer) provided by the kit, tissue was homogenized to a fluid state. Each tube received 350 µl of CTL buffer, and tissues were crushed again, pushing the pestle against the tissue and the wall of the microcentrifuge tube. This was done for approximately 20 min or until crushing the exoskeleton resulted in a visibly homogenous lysate and breakdown of the exoskeleton was no longer audible. Sample tissue that became too compacted at the bottom of the tube (Fig. 3C) was resuspended in the buffer using a pair of autoclaved forceps and additionally crushed using an autoclaved pestle. The head and body tissues appeared substantially darker in color compared to the leg tissues (Fig. 3, C–D and E). When tissue types appeared homogenous, 25 µl of Proteinase K (included in the kit; Omega Bio-Tek SKU#: PROK-50) was added to digest proteins and samples were mixed by vortexing in five short, repeated bursts. The samples were incubated at 60°C for 1 h and then incubated at 37°C in a dry bath overnight. Incubation time varied between 8 and18 h, and on average a 12-h period corresponded with the highest DNA yield.

After incubating overnight, 350 µl of a freshly prepared 24:1 chloroform:isoamyl alcohol solution was added to each tissue sample’s tube. Sample tubes were then centrifuged at 10,000 × *g* for 2 min at 20°C (room temperature). Centrifugation resulted in phase separation. The upper aqueous layer of each sample was transferred, 100 µl at a time, into a new sterile microcentrifuge tube. The excess exoskeleton mass is removed here, as transfer of the upper aqueous phase failed to collect the heavier components of the exoskeleton. On average, around 500 µl of aqueous phase was transferred from the body segments and 250 µl from head and leg samples, respectively, though some variability is expected. One volume of BL buffer and 2 µl of RNase A were added to each sample and mixed by vortexing. The samples were then placed in a dry bath and incubated at 70°C for 10 min. To prepare for the last elution step of the extraction protocol, 150 µl of the elution buffer provided in the kit was left to incubate at 70°C in a separate microcentrifuge tube. After incubation, one volume of 100% molecular grade ethanol was added to each extraction tube and mixed by vortexing. A sample volume of 500 µl was transferred into a HiBind DNA Mini Column inserted into a 2 ml collection tube, both provided by the kit. Samples were centrifuged at max speed (21,100 × *g*) for 1 min. The filtrate was then discarded, the remaining sample volume was added to the same mini column, and the previous step was repeated until all the samples had gone through the column. Each sample received 500 µl of HBC buffer and was centrifuged at max speed for 1 min.

The filtrate was discarded, and the column was washed twice using the DNA wash buffer. For each wash, 700 µl of DNA wash buffer was added to each sample, centrifuged at max speed, with the filtrate discarded after each time. After DNA washing, the samples were dried by centrifugation for an additional 2 min at maximum speed. The collection tubes were then discarded and each mini column containing DNA was transferred to a new, sterile 1.5 ml microcentrifuge tube. About 50 µl of the previously heated elution buffer was added to the center of each mini column. Samples were incubated for 5 min per the manufacturer’s recommendation to aid in removing the bound DNA from the column membrane. Next, samples were centrifuged at maximum speed for 1 min. The filtrate was then added back into the column for a second elution. After the second elution, samples were incubated for an additional 5 min and centrifuged under the same conditions. This helped to maximize DNA concentration, as reusing the eluate significantly reduces sample volume.

### Genomic DNA clean-up and size selection

Omega Biotek Mag-Bind Total Pure NGS Beads (Cat. No. M1378) were used to remove short fragments of DNA and remaining DNA extraction buffers. Remaining alcohol-based buffers can distort DNA quantitation, leading to inaccuracies in downstream analyses. This is a crucial step for accurate DNA quantitation and size selection for DNA used in NGS sequencing [23]. Magnetic bead cleanup also aided in removing contaminants, such as residual salts, enzymes, and other proteins that can interfere with downstream applications [23]. A 1.8X volume of magnetic beads (90 µl of resuspended beads for every 50 µl of sample) was added to each sample and briefly vortexed with five short bursts. Samples were left to mix for 5 min before placement onto a magnetic bead separation rack compatible with our 1.5 ml sample tubes. After magnetic beads and DNA were bound to the magnetic rack, the filtrate for each tube was disposed of and each sample was washed twice with 250 µl of 70% ethanol and then eluted in 30 µl of nuclease free water.

### Quantitation, length and quality analysis, and storage

Concentration was determined immediately following genomic DNA clean-up. Nanodrop spectrophotometry (ThermoFisher Scientific ND-2000) was used to calculate 260/230 and 260/280 ratios and determine sample purity. An Invitrogen Qubit 4.0 fluorimeter with dsDNA HS Assay Kit (Q32851) was also utilized to measure accurate DNA concentration. The Agilent TapeStation 4200 automated electrophoresis system with a Genomic DNA ScreenTape (Screen Tape No. 5067- Reagent No. 5366) was used to analyze each DNA sample to assess DNA size. DNA samples were diluted with deionized water to meet the kit requirement of DNA concentrations between 10 and 100 (ng/μl). Quantitated DNA samples were stored at −20°C in parafilm-sealed microcentrifuge tubes until sequencing.

### Long-read sequencing using nanopore MinION and analysis using EPI2ME workflows

The Rapid Sequencing Kit V14 (SQK-RAD114) by Oxford Nanopore was used to create DNA libraries from head tissue from a wild caught *C. georgiana* specimen. From the same individual specimen, we also prepared libraries from head, body, and legs with the Ligation Sequencing Kit without the optional fragmentation step using (SQK-LSK110) The flowcell used was FLO-MIN106. Approximately 100 ng of DNA was prepared according to the manufacturer’s protocol and loaded onto a MinION Flow Cell. Samples were sequenced on the Oxford Nanopore Technologies MinION (Mk1b) to generate basecalled reads (with default fast basecalling in MInKNOW, which uses Guppy/Dorado) in a FASTQ format. The pre-built wf-alignment and wf-bacterial-genomes workflows in EPI2ME were utilized for alignments and reference-guided assemblies respectively using *Appalachioria falcifera* as the reference. Mitochondrial DNA from three tissue types using the ligation sequencing datasets were also aligned with Clustal multiple sequence alignment to detect variations. Lastly, also using EPI2ME wf-alignment workflow we aligned basecalled reads from body DNA with an available whole genome assembly from millipede *Helicorthomorpha holstii* [8].

### Mitochondrial genome assembly, alignment, and phylogenetic tree construction

The mitochondrial genome of *A. falcifera* (GenBank JX437063.1) was selected as the reference since it was the most closely related mitochondrial genome available (both are classified in the same subfamily, Rhysodesminae). Dotmatics Geneious Prime Version 2023.2.1 was utilized for sequencing data analysis, with Minimap2 Version 2.10 employed to align preprocessed sequencing reads from *C. georgiana* to the *A. falcifera* reference mitochondrial genome. Alignment parameters, such as seed length and mapping quality thresholds, were adjusted to optimize both accuracy and sensitivity. Upon generating the consensus sequence and mitochondrial map, the quality scores of the sequencing reads and the entire mitochondrial genome were rigorously evaluated to ensure accuracy and completeness. The annotation process involved the identification of tRNA genes, rRNA genes, and protein-coding genes within the mitochondrial genome. All detected mutations and sequence variations were documented, and tRNA structures were predicted using the Turner 2004 energy model [24] within Geneious Prime, providing a comprehensive characterization of the mitochondrial genome.

Phylogenetic analysis was also performed using the complete mitochondrial genome of *C. georgiana*. Reference-guided assemblies of body, head, and leg samples from a single millipede were aligned using Clustal OMEGA in Geneious Prime to generate a consensus sequence. Sequence annotations were transferred from 14 publicly available mitogenomes in cases where sequences had a similarity score of >75% (Fig. 7). Phylogenetic tree construction utilized the Geneious Tree Builder tool within Geneious Prime to construct the tree in a pairwise alignment of all sequences. The Tamura–Nei genetic distance model was used to determine species divergence in conjunction with a neighbor-joining building method [25]. The generated phylogenetic tree (Fig. 8) comprises 15 total millipede species within the order Polydesmida, including our annotated *C. georgiana* mitogenome. The centipede species *Strigamia maritima* was also used as an outgroup.

## Results

### High-purity DNA extracted from tissue types: Legs, head, and body

We dissected millipede specimens to extract genomic DNA from three distinct tissue types—legs, head, and body (Table 1). We analyzed six wild-caught *C. georgiana* specimens, from six replicates. Starting sample mass for legs ranged from 28 to 56 µg for an average of ∼45 legs per sample. Head tissue samples had higher weight ranging from 64 to 89 µg. Tissues for the body segments had the highest weight at 235–330 µg, however, these also had the largest amount of non-soft tissue exoskeleton mass. For each sample, we tested extracted DNA quality using UV absorbance ratios. All samples met the sample purity criteria exceeding threshold values of 1.8 and 2.0, for ratios 260/280 and 260/230, respectively (Table 1).

**Table 1.** UV Absorbance and DNA concentration obtained from *C. georgiana* tissues. Tissue weight, UV absorbance, and DNA concentration of head, body, and leg tissues from six *C. georgiana* specimens presented in this study. The 260/280 ratios in all samples indicate pure total DNA samples. We found UV DNA concentrations to vary widely and were only used to broadly estimate true DNA yield.

Sample	Tissue Weight (g)	UV Absorbance Ratio: 260/280	UV Absorbance Ratio: 260/230	UV DNA Concentration (ng/μl)
*Legs*				
1	0.028	2.04	2.31	617.1
2	0.048	1.95	2.24	250.3
3	0.037	2.05	2.23	618.5
4	0.056	2.01	2.21	176.6
5	0.054	2.07	2.22	311.3
6	0.042	1.96	2.02	79.8
*Head*				
1	0.064	2.06	2.07	105.2
2	0.089	1.99	2.27	641.0
3	0.081	2.01	2.07	102.8
4	0.077	2.08	2.22	296.8
5	0.084	2.10	2.13	99.6
6	0.068	2.05	2.14	204.5
*Body*				
1	0.282	1.99	2.27	830.4
2	0.330	1.89	2.23	257.2
3	0.235	1.98	2.28	888.8
4	0.298	1.84	2.46	98.5
5	0.305	1.95	2.08	101.1
6	0.273	1.93	2.24	90.9

For accurate DNA concentration and yield calculations, we used fluorometry instead of UV absorbance-based concentrations. We note that while fluorometric quantitation did not provide information on sample purity it was more reliable compared to UV absorbance for accurate quantitation, a critical measurement for DNA sequencing. Leg tissues, which contained the least amount of starting weight, produced an average of 1.78 µg per sample (Table 2). Legs also had the highest average DNA yield extracted per gram of starting tissue used with an average of 44.57 µg/g, while head tissue had an average of 40.59 µg/g (Table 2). As expected, body rings produced the highest total DNA yield averaging 5.54 µg with a maximum of 14.25 µg (Table 2). However, we observed that the fluorometric concentration in body samples varied considerably between specimens, ranging from a maximum of 475 ng/µl to a minimum of 20.7 ng/µl. Factoring in starting tissue mass, we observed that specimens 5 and 6 have low net DNA yield. This resulted in body samples having the lowest average DNA yield, with an average of 20.2 µg/g. Excluding specimens 5 and 6, which appear to have low DNA yield across tissue types, the body average was 28.9 µg/g, still considerably lower than other tissue types.

**Table 2.** Summary of DNA extraction by tissue type. Fluorometric DNA concentrations and total DNA weight obtained for head, body, and leg tissues from six *C. georgiana* specimens presented in this study. Fluorometric DNA concentrations were found to have higher accuracy than our UV DNA concentration estimates, making this method of quantitation ideal for downstream analysis.

Sample	Tissue Weight (g)	Fluorometric DNA Concentration (ng/μl)	Total DNA Yield (μg)	DNA Yield per Gram of Dissected Tissue (μg/g)
*Legs*				
1	0.028	60.2	1.806	64.50
2	0.048	74.1	2.223	46.31
3	0.037	120	3.600	97.30
4	0.056	50.3	1.509	26.95
5	0.054	27.8	0.834	15.44
6	0.042	23.7	0.711	16.93
*Head*				
1	0.064	191	5.73	89.53
2	0.089	187	5.61	63.03
3	0.081	88.2	2.646	32.67
4	0.077	56.9	1.707	22.17
5	0.084	27.7	0.831	9.893
6	0.068	59.6	1.788	26.29
*Body*				
1	0.282	475	14.25	50.53
2	0.330	160	4.8	14.55
3	0.235	308	9.24	39.32
4	0.298	115	3.45	11.58
5	0.305	29.7	0.891	2.92
6	0.273	20.7	0.621	2.27

### Extracted DNA average lengths ranged between 12 and 25 kb

**Figure 4.**
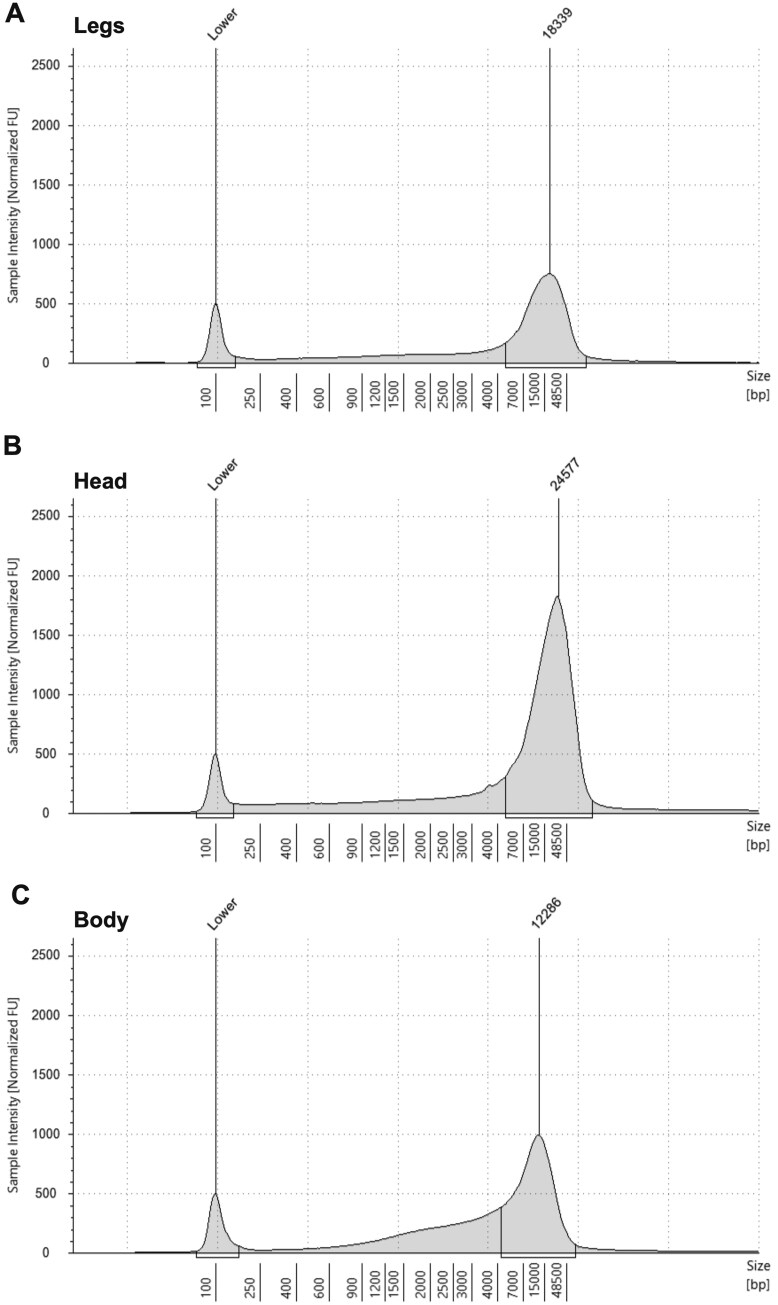
Analysis of DNA size across tissue types using automated electrophoresis. Analysis of DNA length using the Agilent TapeStation 4200 Automated Electrophoresis System. Average DNA sequence length from leg tissue (A) was 18.3 kb. DNA from head tissue (B) had the longest sequence length of our samples, averaging 24.5 kb. Comparatively, sequences from body tissue were most likely cleaved during crushing and had the lowest average sequence length of 12.2 kb (C)

To measure DNA size, we used automated electrophoresis with genomic DNA ScreenTape. We show representative electropherograms from three tissue types generated using Agilent TapeStation Analysis Software version 4.1.1, with DNA lengths of 18.3, 24.6, and 12.3 kb for legs, head, and body tissues, respectively (Fig. 4). For body tissues, we observed elevated levels of DNA in the 2000–7000 bp length range as compared to legs and head tissue (Fig. 4C).

### ONT long-read sequencing and analysis of tissue-specific mitochondrial DNA

**Figure 5.**
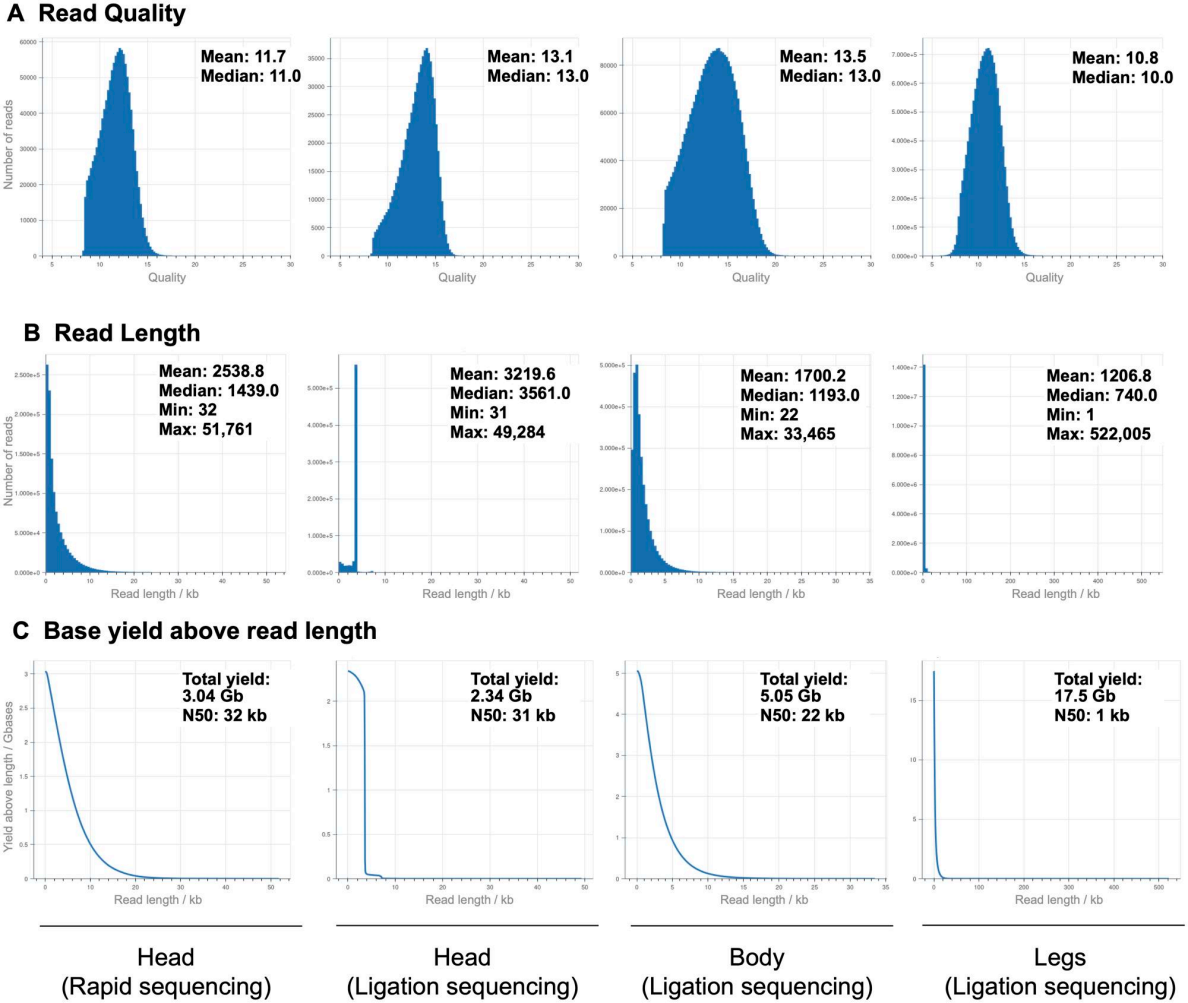
Quality metric data for obtained sequences. Charts depicting read quality, length, and base yield above read length for each of the following: head tissue samples using the rapid sequencing kit, head tissue samples using the ligation sequencing kit, body tissue samples using the ligation sequencing kit, and legs using the ligation sequencing kit (left to right). Read quality was highest in the body tissues, while head tissues exhibited the highest mean sequence lengths

**Figure 6.**
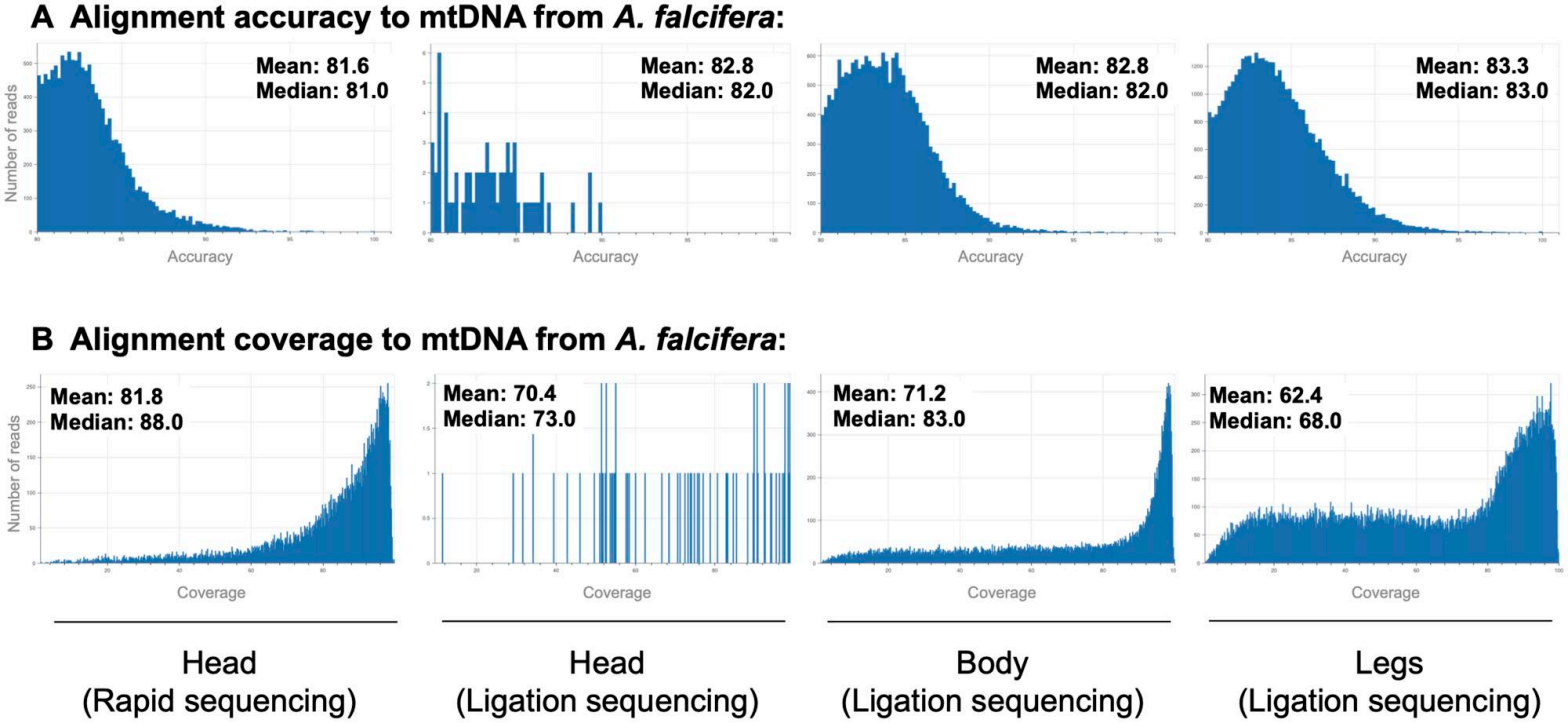
Alignment accuracy to mtDNA for *A. falcifera*. Alignment of basecalled reads to the millipede species *A. falcifera*. Quality varied greatly according to tissue type, with body tissue samples having the highest consistent quality. Tissue from leg DNA may be suitable for less intensive analyses, particularly in cases where soft tissue dissection is impractical

Using DNA sample for millipede head tissue, ONT MinION long-read sequencing with a rapid sequencing kit produced 3.5 Gb of sequencing data, of which 3.04 Gb passed the minimum Q score threshold of 8. From a total of 1.45 million reads the estimated N50 score was 4.74 kb (Table 3). From the basecalled data, we also detect reads as long as 20 kb (Table 3). Among the outlier data, we found that among the longest 1% of reads from the total dataset, 26.71 Mb of aggregated reads were 20.5–28.5 kb in length (Table 3). From the rapid kit data, we observed a mean read quality of 11.7, mean read length of 2538.8 (maximum length over 51 kb), and an N50 of 32 kb (Fig. 5). The same DNA sample was also analyzed using the ligation sequencing kit and we detect higher mean read quality and length of 13.1 and 3219.6. The two datasets from the head tissue are largely comparable, although the rapid kit produced a high yield likely due to an abruptly terminated sequencing run. Body and leg tissue samples from the same specimen were also sequenced using the ligation sequencing kit. We observed the highest read quality for the dissected body tissue sample, although the read length and N50 is lower than the head tissue (mean length of 1700.2 and N50 of 22 kb). With tissue from legs while we had a very high yield, from the mean read length and base yield above read length histograms it is clear that the sample was fragmented resulting in aberrant data. This is likely due to long term storage of purified genomic DNA prior to sequencing. We aligned basecalled reads to the *A. falcifera* mtDNA which produced consistent accuracy scores between 81 and 83 (Fig. 6). Only 73 reads from ligation sequencing of the head DNA aligned to the target, as compared to several thousands of aligned reads from the other samples. Low mean coverage in legs is likely to have resulted from issues with fragmentation (Fig. 6). We conducted reference-guided mtDNA assembly from each sample, and specifically compared the three tissue types to detect variations (Supplementary Fig. S2). Overall, we observe that despite broad similarities, several localized variations are detected. Despite the mixed quality in our compared datasets, this strategy for detecting tissue-specific variants may be valuable for future analysis using dissected tissue samples from the same specimen.

**Table 3.** ONT MinION Rapid Sequencing run statistics. Feasibility test for long-read sequencing using head tissue DNA as input. MinION Mk1b using flow cell FAS80583 and kit SQK-RAD004. Control software MinKNOW version 22.12.7

*Data produced*	41.3 GB	
*Estimated bases*	3.5 Gb	
*Reads generated*	1.48 million	
*N50 (estimated)*	4.74 kb	
*Basecalling (minimum Q score 8)*	3.04 Gb pass 633.73 Mb fail	Basecaller: Guppy version 6.4.6 (Fast model 450 bps)
*Outliers (longest 1% strands)*	20.5–28.5 kb	26.71 Mb aggregated reads
	28.5–36.5 kb	3.33 Mb aggregated reads

### Mitogenomic annotation and phylogenetic analysis

**Figure 7.**
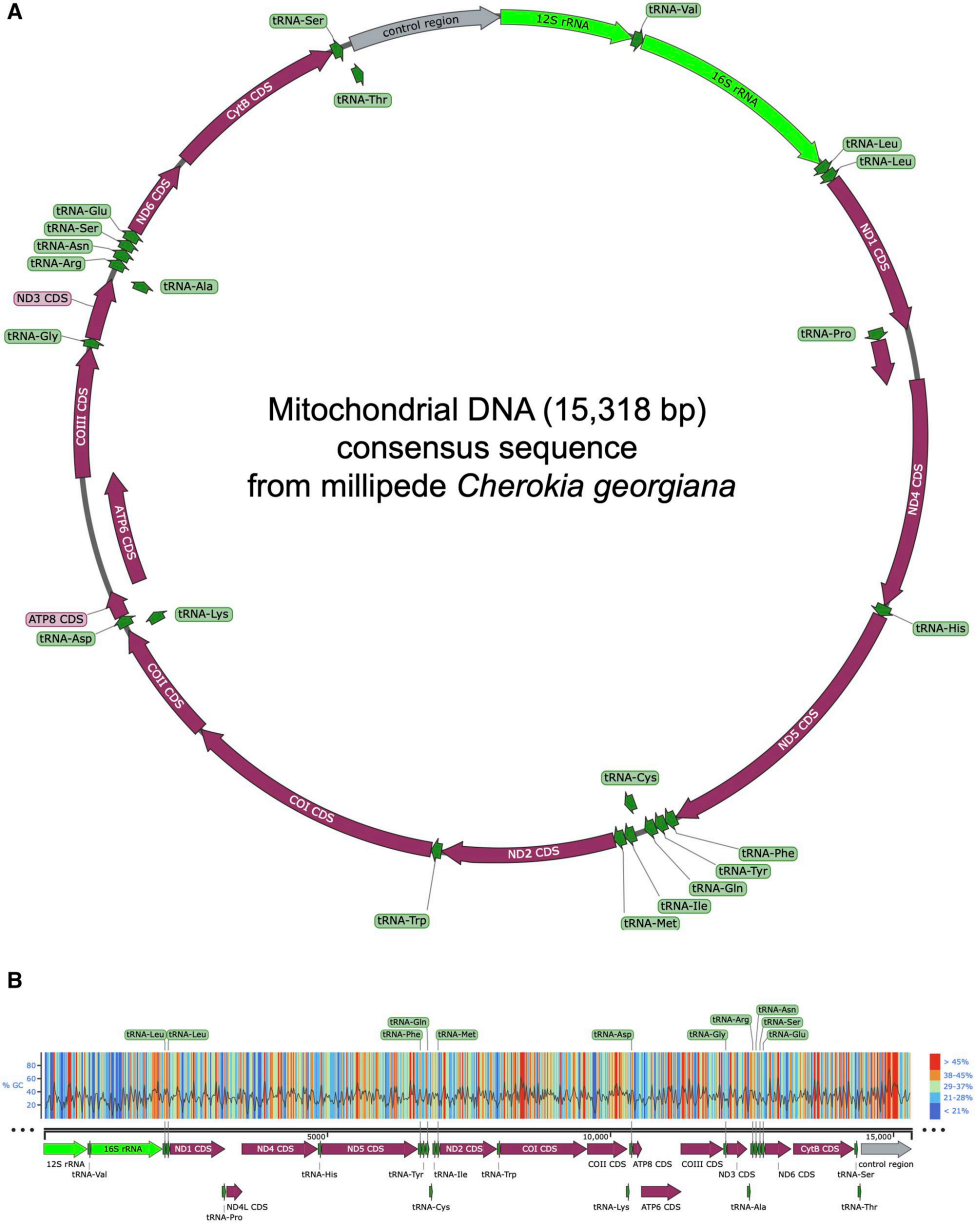
Annotated mitochondrial map of *C. georgiana*. Circularized overview of the *C. georgiana* mitogenome with notes on annotation. Annotations for predicted genes and tRNA portions were transferred from the *A. falcifera* and the mitogenomes of 13 other millipede species in sequences found to be at least 75% similar. The mitochondrial map depicted here is the result of a generated consensus sequence using data for all three tissue types

**Figure 8.**
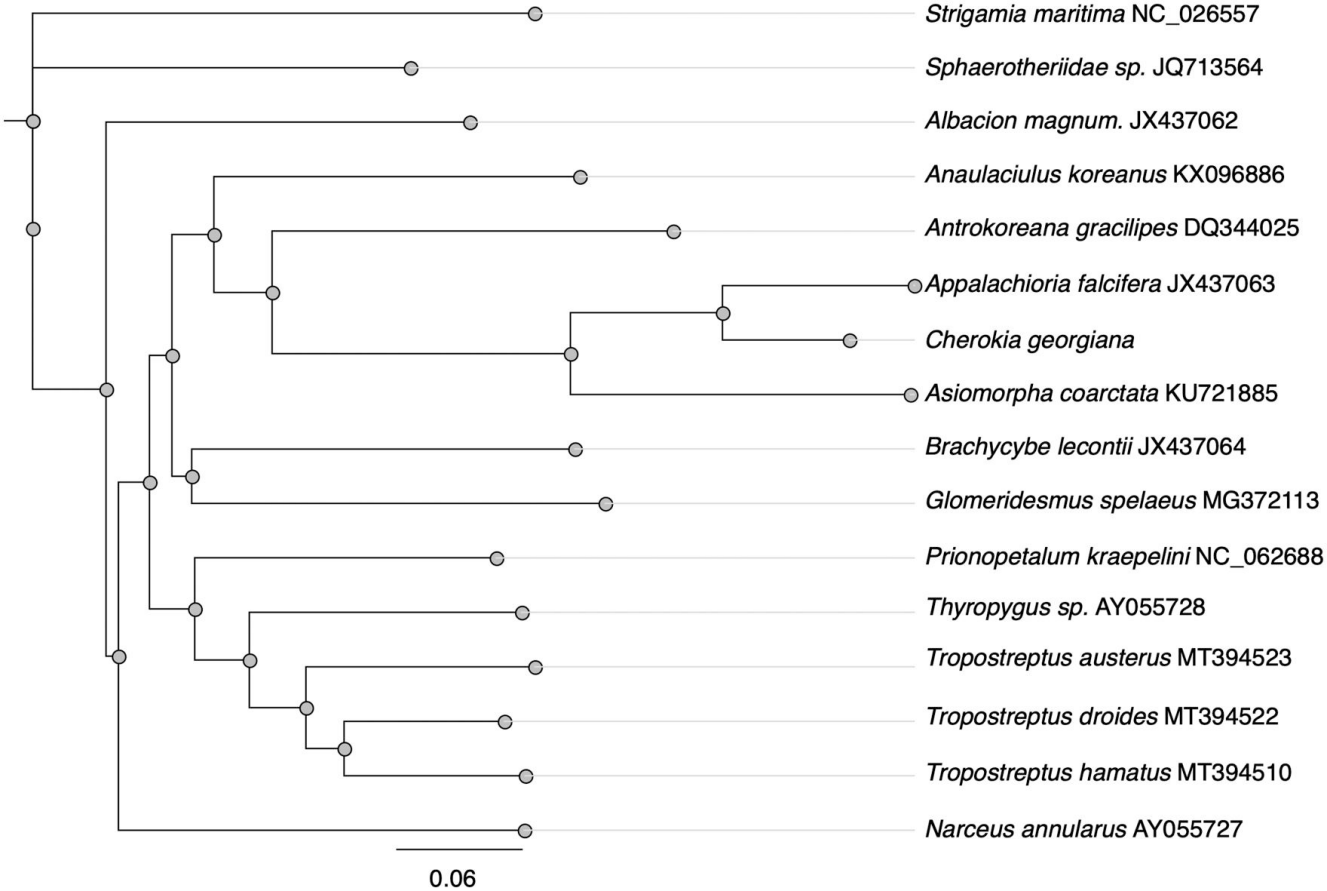
Phylogenetic tree construction using full mitogenomes. Phylogenetic tree showing the evolutionary position of *C. georgiana* based on the complete mitogenomes of 14 other millipede species, using the centipede *S. maritima* as an outgroup. A branch length of 0.06 represents an estimated 6% evolutionary divergence between nodes. The distribution of several millipede species within the order Polydesmida can be seen here, with *A. falcifera* identified as the closest relative to *C. georgiana*

Annotation of the mtDNA for *C. georgiana* was performed through detailed gene prediction analyses, including the identification of protein-coding genes, tRNA genes, rRNA genes, and other functional genomic features. Annotations were transferred from its closest available relative, *A. falcifera*, as well as 13 additional millipede species within the same order. Annotations were transferred only in sequences identified to at least 75% similar to a reference. The majority of our annotations were derived from *A. falcifera*, as it is the closest relative to *C. georgiana* with a publicly available mitogenome. The resulting alignment map provided a depiction of sequence similarity between several species within the Polydesmida, enabling comparisons and identification of conserved regions. The estimated size of *C. georgiana*’s mitochondrial genome was approximately 15,318 base pairs. Here, we present a complete annotation of *C. georgiana’*s mitogenome to be used in further phylogenetic analysis (Fig. 7). As expected, the mitogenomes of *C. georgiana* and *A. falcifera* were found to be the most closely related of all species used in this study (Fig. 8).

### Alignment with whole genome assembly of *H. hosltii*

While our nanopore sequencing methodology had several limitations into terms of accuracy, depth, and quality, we were interested in broader information that can be collected using the sequencing dataset. We aligned our highest quality reads from the body tissue DNA to the high-quality whole genome assembly from *H. holstii*. From nearly 3 million reads, only 5.7% aligned to our target (Supplementary Fig. S3). We detect a mean accuracy of 78.3 and low mean coverage of 24.1. While the data quality and approach limit our ability to interpret this alignment much further, as a quick diagnostic approach this can potentially be used as a tool for measuring species divergence.

## Discussion

In this study, we present a sample dissection protocol and a procedure for DNA extraction from the millipede species, *C. georgiana,* for nanopore DNA sequencing. Nanopore DNA sequencing is rapidly improving and it uniquely offers some key advantages to more commonly applied methodologies, such as lower overall cost, potential for field-based analysis, option for direct, PCR-free genomic DNA sequencing, and pre-built analysis workflows. The utility of nanopore sequencing on the ONT MinION platform is limited compared to other technologies—it is not designed to sequence a large genome with high accuracy and depth. However, sequencing on an ONT MinION serves as a means to assess sample quality and evaluate specific sequencing parameters prior to more robust methodologies for whole genome sequencing and assembly. As a long-read sequencing technology, it is a prerequisite to prepare high quality DNA samples with minimal fragmentation for optimal sequencing results. This can be challenging for certain species where morphological characteristics make high quality DNA extraction problematic. Furthermore, getting adequate quality DNA from specific tissue types may present more complications. Here we provide a method for DNA extraction from millipedes after sample dissection to isolate DNA from head, body, and legs. We also include analysis of the DNA from different tissue types using the ONT MinION, perform alignment and assembly with a reference mitochondrial DNA, and analyze tissue-specific mtDNA variations. We also note that data collected using our methodology can be used to analyze any abundant DNA and is not limited to mtDNA analysis. However, since mtDNA is commonly used for phylogenetic analysis, our approach elucidates a workflow from sample to assembled mitogenome to phylogenetic tree prepared entirely in-house in a relatively short time.

The exoskeleton of arthropods is scarce as a DNA source and negatively impacts DNA extraction [26]. Chitin found in the exoskeleton can also lead to DNA yield overestimation [15]. Dissection prior to DNA extraction also enables removal of the specimen digestive tract, mitigating contamination. The digestive tract contains food particles (plant detritus and soil organic matter) and attendant bacterial and fungal decomposers [27]. The digestive tract can also include parasitic or commensal nematodes and other eukaryotes that introduce foreign DNA sequences [3, 28]. Genomic DNA from specimen legs, head, and body tissue were extracted and isolated to remove short fragments.

We were able to extract high-purity DNA samples from all three tissue types based on UV absorbance ratios (Table 1). Furthermore, total DNA yield from all tissue types is sufficient for downstream NGS applications. For several applications, collecting specimen legs, a straightforward procedure, can serve as an adequate source of DNA. If higher total DNA amount is required, then head and body samples can both serve as an abundant DNA source (Table 2). We observed maximum DNA yield per source tissue mass for the head tissue and found it to be the most viable DNA source for sequencing data (Table 2). The highest total DNA yield was from the body, the tissue source with the highest mass (Table 2).

All three tissue types can be used on the Illumina sequencing platform for applications such as whole genome sequencing and whole transcriptome/mRNA sequencing. Since Illumina sequencing produces DNA fragment libraries in the 150–600 bp length, DNA length is not a concern. Platforms such as NovoSeq 6000 and NovoSeqX are ideal for whole genome projects for millipedes, where genome sizes of sequenced species range between 149 and 612.5 Mb [8]. We observed DNA lengths in the 15–25 kb range with all tissues, which can be useful for targeted long read sequencing applications. While higher DNA lengths are required for whole genome sequencing on platforms like ONT and PacBio, specific applications such as mitochondrial DNA sequencing for phylogenetic and evolutionary analysis are feasible. In our test ONT run on a MinION using the rapid sequencing protocol, we obtained an estimated N50 value of 4.74 kb, with maximum read length greater than 20 kb (Table 3). Sequencing and assembly of short-to-medium length target regions are achievable using any of the tissue samples.

We also performed ONT MinION sequencing on the same millipede specimen using ligation sequencing without fragmentation. Here we note some differences in data quality, particularly in DNA extracted from head and legs, which are most likely due to interrupted sequencing and long-term storage prior to sequencing, respectively (Fig. 5). Regardless, we were able to analyze these datasets measuring read quality, length, and N50 scores (Fig. 5). It is interesting to note that N50 scores obtained post-alignment significantly exceed the estimated N50 as detected from primary sequencing statistics. Upon alignment with the mitochondrial reference DNA from *A. falcifera*, we also compare alignment statistics from each tissue-type (Fig. 6). We note that our best quality dataset is from the body DNA, however, this is also the most skill-intensive dissection step. While our data from legs is suboptimal, sequencing extracted DNA promptly with minimal storage will likely improve the quality of output data. Considering that it provided the largest amount of DNA for tissue mass (Table 2), and that it is straightforward to isolate, using leg DNA may be useful for most applications. We also do not see any major advantage in using ligation sequencing as compared to rapid sequencing from the perspective of targeting alignment to an abundant DNA. From our mtDNA alignment, we observe that across tissues there are localized variations (Supplementary Fig. S2). Building a consensus sequence for the *C. georgiana* mtDNA, we present the fully assembled mitogenome of length 15,318 bp (Fig. 7). Using the mtDNA sequence data, we also generate a phylogenetic tree (Fig. 8).

We identify avenues to further improve DNA extraction from millipedes. Separating each body segment permits easier tissue homogenization, however, during this process DNA fragmentation is observed in our electrophoresis data (Fig. 4). This limits our DNA product viability in terms of long read whole genome sequencing. Further optimization of tissue homogenization methods is required to achieve DNA in the HMW size range of 50–200 kb. For applications like Hi-C sequencing that require soft-tissue, a high DNA yield after scraping soft tissue from body segments will be necessary.

Overall, our dissection strategy and DNA extraction benchmarks provide a robust starting point for a wide range of sequencing applications. While our protocol was tested with *C. georgiana*, we anticipate that DNA from other millipede species with similar morphology can be extracted using our procedure. Several interesting functions in the remarkable order Polydesmida remain to be genetically described and we anticipate that the method described here will stimulate further genomic analysis in this field.

## Supplementary Material

bpaf042_Supplementary_Data

## Data Availability

FASTQ sequencing files are available at NCBI SRA using accession number PRJNA1262952.
