# Supplementary material for: Tissue-specific DNA isolation from dissected millipedes for nanopore sequencing
Source: Biol Methods Protoc. 2025 May 28;10(1):bpaf042. doi: 10.1093/biomethods/bpaf042 (PMC12202871; doi:10.1093/biomethods/bpaf042)
Supplement: bpaf042_Supplementary_Data [file bpaf042_supplementary_data.zip › bpaf042_Supplementary_Data.html]

CG27Head\_lig-seq 1 TGAAAATAAATTTTATGTTGTCTTTATAATATTTGATTATGGTATTTTATCTTGCTTTTGATGAAGTTTATATGAGAAGTTTAATAGCCTAGAGGGCTTATTTTTAGTAAAAGAAATTAT  
CG27Body\_lig-seq 1 TGAAAATAAATTTTATGTTGTCTTTATAATATTTGATTATGGTATTTTATCTTGCTTTTGATGAAGTTTATATGAGAAGTTTAATAGCCTAGAGGGCTTAATTTTAGTAAAAGAAATTAT  
CG27Legs\_lig-seq 1 TGAAAATAAATTTTATGTTGTCTTTATAATATTTGATTATGGTATTTTATCTTGCTTTTGATGAAGTTTATATGAGAAGTTTAATAGCCTAGAGGGCTTAATTTTAGTAAAAGAAATTAT  
  
CG27Head\_lig-seq 121 TTTTGATGTTAATATTAAATGAGTTTTAATTTTAAGCTAATGTTGTGCCAGCGGCTGCGGTTATACAACTTAAGGTGTGAGATTTGGTGGTAAAAGAAAGTGTTATTTTTTGTTAGATGA  
CG27Body\_lig-seq 121 TTTTGATGTTAATATTAAATGAGTTTTAATTTTAAGCTAATGTTGTGCCAGCGGCTGCGGTTATACAACTTAAGGTGTGAGATTTGGTGGTAAAAGAAAGTGTTATTTTTAGTTAGATGG  
CG27Legs\_lig-seq 121 TTTTGATGTTAATATTAAATGAGTTTTAATTTTAAGCTAATGTTGTGCCAGCGGCTGCGGTTATACAACTTAAGGTGTGAGATTTGGTGGTAAAAGAAAGTGTTATTTTTAGTTAGATAG  
  
CG27Head\_lig-seq 241 TAGTGGGAAGATTAGTTAGTTTTTGGTAGAATTGTTACTTATGTAGTTCTTATTTT-TATATA-GGGATTAATTATTTATTTTGGTGAAATTATAAACTAGGATTAGATACCCTATTATT  
CG27Body\_lig-seq 241 TGGGGAAAAGATTAGTTAGTTTTTGGTAGAATTGTTACTTATGTAGTTCTTATTTTCTATATATAGGATTAATTATTTATTTT-GTGAAATTATAAACTAGGATTAGATACCCTATTATT  
CG27Legs\_lig-seq 241 TAGTG--AAGATTAGTTAGTTTTTGGTAGAATTTTTACTTATGTAGTTCTTATTTTCTATATATAGGATTAATTATTTATTTT-GTGAAATTATAAACTAGGATTAGATACCCTATTATT  
  
CG27Head\_lig-seq 359 TTTATAAAAATGTTTACCGTAGAATTAGGGGTATTATTTGAAATTAAAATGATTTGGCAGCGTGTTGTCTCATCAGAGGAACCTGAAGTTGAGTCGAAACCCCGCGAGTAGTTAATCTTT  
CG27Body\_lig-seq 360 TTTATAAAAATGTTTACCGTAGAATTAGGGGTATTATTTGAAATTAAAATGATTTGGCGGCGTGTTGTCTCATCAGAGGAACCTGAAGTAGAGTCGACAACCCGCGAGTAGTTAATCTTT  
CG27Legs\_lig-seq 358 TTTATAAAAATGTTTACCGTAGAATTAGGGGTATTATTTGAAATTAAAATGATTTGGCGGCGTGTTGTCTCATCAGAGGAACCTGAAGTAGAGTCGACAACCCGCGAGTAGTTAATCTTT  
  
CG27Head\_lig-seq 479 TCTATTAGTTTGTATATCTCCGTCGAAAGAAGAATTTTTATAAAATATCTTCTGATATTAAGAAAGAAAGCTAAGACAGGTCAAGGTGCAGCTTATGAAGAGGATTCTTTGGGTTACAAT  
CG27Body\_lig-seq 480 TCTATTAGTTTGTATATCTCCGTCGAAAGAAGAA-TTTTATAAAATATCTTCTGATATTAAGAATGAAAGCTAAGACAGGTCAAGGTGCAGCTTATGAGAAGGATTCTTTGGGTTACAAT  
CG27Legs\_lig-seq 478 TCTATTAGTTTGTATATCTCCGTCGAAAGAAGAA-TTTTATAAAATATCTTCTGATATTAAGAGATAAAGCTAAGACAGGTCAAGGTGCAGCTTATGAGAAGGATTCTTTGGGTTACAAT  
  
CG27Head\_lig-seq 599 TTTTGTTGGAAG-ACGGATTAGAACAAAGGATTGGTTTTTTGAAGGTGGATTTGTAAGTAATTA-TTTTTATAGAATTATTAAATGAAGGGTGGAATAACATGTGTACATATTGCCCGTC  
CG27Body\_lig-seq 599 TTTTGTTGGAAGAACGGATTAGAAC-AAGGATTGGTTTTTTGAAGGTGGATTTGTAAGTAATTATTTTTTATAGAATTATTAAATGAAGGGTGGAATAACATGTGTACATATTGCCCGTC  
CG27Legs\_lig-seq 597 TTTTGTTGGAAGAACGGA--AGAAC-AAGGATTGGTTTTTTGAAGGTGGATTTGTAAGTAATTATTTTTTATAGAATTATTAAATGAAGGGTGGAATAACATGTGTACATATTGCCCGTC  
  
CG27Head\_lig-seq 717 GCTCTCGTTATTTTTGAGATAAGTCGTAACATAGTTGATGAACTGGAAAGTGTATCTTTTTGACATATTAGAGTTTATTTTGAATTTTTCCTTTACATTGAAAAGGTCTCTTGTGAGTAA  
CG27Body\_lig-seq 718 GCTCTCGTTATTTTTGAGATAAGTCGTAACATAGTTGATGAACTGGAAAGTGTATCTTTTTGACATATTAGAGTTTATTTTGAATTTTTCCTTTACATTGAAAAGGTCTCTTGTGAGTAA  
CG27Legs\_lig-seq 714 GCTCTCGTTATTTTTGAGATAAGTCGTAACATAGTTGATGAACTGGAAAGTGTATCTTTTTGACATATTAGAGTTTATTTTGAATTTTTCCTTTACATTGAAAAGGTCTCTTGTGAGTAA  
  
CG27Head\_lig-seq 837 TATGATGATTATTTTTTAGGATTTTATTTTAATAATTAGAAAATTTGTTATAAGTAGTTTGAGCGAATTTAGTTGGTGTCTTATAGGGGATTAAGTACTGAGAAGGAATGTTTATTATTT  
CG27Body\_lig-seq 838 TATGATGATTATTTTTTAGGATTTTATTTTAATAATTAGAAAATTTGTTATAAGTAGTTTGAGCGAATTTAGATGGTGTCTTATAGGGGATTAAGTACTGAGAAGGAATGTTTATTA-TT  
CG27Legs\_lig-seq 834 TATGATGATTATTTTTTAGGATTTTATTTTAATAATTAGAAAATTTGTTATAAGTAGTTTGAGCGAATTTAGATGGTGTCTTATAGGGGATTAAGTACTGAGAAGGAATGTTTATTATTT  
  
CG27Head\_lig-seq 957 TTTTAAAATATTAGGTTATTT-TTGCCTTTTGTATTAGGGTTGATCTATGTGGGTTTACGTTTGTTTTTTCTCGATGAACATCGGGCTATTTTTTTTTAGTTAATTCTCGTTGTATAGAG  
CG27Body\_lig-seq 957 TTTTAGAATATTAGGTTATTTAGTACCTTTTGTATTAGGGTTGATTTATGTGGTTTTACGTTTG-TTTTTCTCGATGAACATCGGGCTATTTTTCTTTTA-GAATTCTCGTTGTATAGAG  
CG27Legs\_lig-seq 954 TTTTAGAATATTAGGTTATTTAGTACCTTTTGTATTAGGGTTGATTTATGTGGTTTTACGTTTGTTTTTTCTCGATGAACATCGGGCTATTTTTCTTTAGTTAATTCTCGTTGTATAGAG  
  
CG27Head\_lig-seq 1076 TTAAATAGGGGATTATAGGTATGAAATATTATTCAAGTTGTTTTTTATCTGGTTCTTTAAGAAATTTAATAGTTATAATATTATTATTGGG-GCGATGGATAGGGATTAGCTTATTTATT  
CG27Body\_lig-seq 1075 TTAAATAGGGATTAATAGGTATGAAATATTATTCAAGTTGTTTTTTATCTGGTTCTTTAAGAAATTTAATAGT--TAATATTATTATTAGTGGCGATGAATAGGGATTAGCTTATTTATT  
CG27Legs\_lig-seq 1074 TTAAATAGGGATTAATAGGTATGAAATATTATTCAAGTTGTTTTTTATCTGGTTCTTTAAGAAATTTAATAGT--TAATATTATTATTAGT-GCGATGAATAGGGATTAGCTTATTTATT  
  
CG27Head\_lig-seq 1195 TTTTATATTGTATTAATTATTTGTTGTTTATTTGTTAAGTTAATAGTGCTTGTTTTGATGGAGTTATTATTGAGTAAGATTTTTATATTATTTTTTGA-TTTGTTTTGTTATTGGGGTTT  
CG27Body\_lig-seq 1193 TTTTATATT-TATTTATTAATTGTTGTTTATTTGTTAAGTTAATAGTGCTTGTTTTGATGGAGTTATTATTGAGTAAGATTTTTATATTATTTTTTGATTTTGTTTTGTTATTGGAGTTT  
CG27Legs\_lig-seq 1191 TTTTATATT-TATTTATTAATTGTTGTTTATTTGTTAAGTTAATAGTGCTTGTTTTGATGGAGTTATTATTGAGTAAGATTTTTATATTATTTTTTGATTTTGTTTTGTTATTGGAGTTT  
  
CG27Head\_lig-seq 1314 TATTGTAAATTTATGATGATTTGTAATAATGATTATATTAGTAATATTTTTGTTAAGGAGTTGCATTTTGA-TGGGTACTGGGGGGTGAAA--ACTCTTATGTGAGGTTAATTTTTAATT  
CG27Body\_lig-seq 1312 TATTGTAAATTTATGATGATTTGTAATAATGATTATATTAGTAATATTTTTGTTAAAGAGTTGCATTTTGA-TGGGTACTGGGGGAGGGAGCTTGAATCTTAGATGGGTAA-TTTTAATT  
CG27Legs\_lig-seq 1310 TATTGTAAATTTATGATGATTTGTAATAATGATTATATTAGTAATATTTTTGTTAAAGAGTTGCATTTTGATTGGGTACTGAGTGA-------TATAATATGTGAGGTTAA-TTTTAATT  
  
CG27Head\_lig-seq 1431 AGAATTGATGAATTCGGCAAAATGGATTTCTGACTGTTTAGCAAAGACATTGTTTTTTGTTTT-AATAAAAAATAAGTTCTGTCCACTGCTATTAGGTGAAGGGCTGCAGTATTTTGACT  
CG27Body\_lig-seq 1430 AGAATTGATGAATTCGGCAAAATGGATTTCTGACTGTTTAGCAAAGACATTGTTTTTTGTTTTTAATAGAAAATAAGTTCTGTCCACTGCTATTAGGTGAAGGGCTGCAGTATTTTGACT  
CG27Legs\_lig-seq 1422 AGAATTGATGAATTCGGCAAAATGGATTTCTGACTGTTTAGCAAAGACATTGTTTTTTGTTTT--ATAAAAAATAAGTTCTGTCCACTGCTATTAGGTGAAGGGCTGCAGTATTTTGACT  
  
CG27Head\_lig-seq 1550 GTATGAAGGTAGCATAATCATTAGCTTTTTAATTAGAAGCTGGTATGAAGGTGAAACAGGGGTTTGCTGTGTTTCTTCTATTTTTGAATTTTATTTTTATGTGAAAAATCGTT-TTGGGT  
CG27Body\_lig-seq 1550 GTATGAAGGTAGCATAATCATTAGCTTTTTAATTAGAAGCTGGTATGAAGGTGAAACAGGGATTTGCTGTGTTTCTTCTATTTTTGAATTTTATTTTTATGTGAAAAATCGTTATTGGGT  
CG27Legs\_lig-seq 1540 GTATGAAGGTAGCATAATCATTAGCTTTTTAATTAGAAGCTGGTATGAAGGTGAAACAGGGATTTGCTGTGTTTCTTCTATTTTTGAATTTTATTTTTATGTGAAAAATCGTTATTGGGT  
  
CG27Head\_lig-seq 1669 TAATGGGACAAGAAGACCCTAATAAGTTTTATCTTCTTTTTTAATGCGTGTATAGGTTTAGAGATTAATGTAATTTATTTTTAGTGGATTTGGCTGGGGCGGCATTTTATTTGACATAGA  
CG27Body\_lig-seq 1670 TAATGGGACAAGAAGACCCTAATAAGTTTTATCTTCTTTTTTAATGCGTGTATAGGTTTAGAGATTAATGTAATTTATTTTTAGTGGATTTGGCTGGGGCGGCATTTTATTTGACATAGA  
CG27Legs\_lig-seq 1660 TAATGGGACAAGAAGACCCTAATAAGTTTTATCTTC-TTTTTAATGCGTGTATAGGTTTAGAGATTAATGTAATTTATTTTTAGTGGATTTGGCTGGGGCGGCATTTTATTTGACATAGA  
  
CG27Head\_lig-seq 1789 TTTATTTTTAACTTTATATGGCGAAAATTTAGACCTAGATTGTTTAGATGAGTGTTAAATTACTGTAGGGATAACAGCGTGATTTTTTTTGAGAGTTCTAATTGACAATAAAGTTTGCGA  
CG27Body\_lig-seq 1790 TTTATTTTTAACTTTATATGGCGAAAATTTAGACCTAGATTGTTTAGATGAGTGTTAAATTACTGTAGGGATAACAGCGTGATTTTTTTTGAGAGTTCTAATTGACAATAAAGTTTGCGA  
CG27Legs\_lig-seq 1779 TTTATTTTTAACTTTATATGGCGAAAATTTAGACCTAGATTGTTTAGATGAGTGTTAAATTACTGTAGGGATAACAGCGTGATTTTTTTTGAGAGTTCTAATTGACAATAAAGTTTGCGA  
  
CG27Head\_lig-seq 1909 CCTCGATGTTGGATTAAGAAGAGCATTGGCGAAGTAGTTGGTGTTTTTGGTCTGTTCGACCAGTAGAATCTTACATGATCTGAGTTCAAACCGGCGTGAGCCAGGTTGGTTTCTATCTTT  
CG27Body\_lig-seq 1910 CCTCGATGTTGGATTAAGAAGAGCATTGGCGAAGTAGTTGGTGTTTTTGGTCTGTTCGACCAGTAGAATCTTACATGATCTGAGTTCAAACCGGCGTGAGCCAGGTTGGTTTCTATCTTT  
CG27Legs\_lig-seq 1899 CCTCGATGTTGGATTAAGAAGAGCATTGGCGAAGTAGTTGGTGTTTTTGGTCTGTTCGACCAGTAGAATCTTACATGATCTGAGTTCAAACCGGCGTGAGCCAGGTTGGTTTCTATCTTT  
  
CG27Head\_lig-seq 2029 TA--TTTTTTGGGTGTGGTATTAGTACGAAA--ATTGTATCGTAGAAATTG-TTTTTTAATGATGCGGTTTAATTTGATTAGTTTGGCAGAGTAGTGCATCGACGTTAGAATTCGAATAT  
CG27Body\_lig-seq 2030 TATTTTTTTTGGGTGTGGTATTAGTACGAAAGGATTGTATCGTAGAAATTGTTTTTTTAATGATGCGGTTAATTTAGATTAGTTTGGCAGAGTAGTGCATCGACGTTAGAATTCGAATAT  
CG27Legs\_lig-seq 2019 TA-TTTTTTTGGGTGTGGTATTAGTACGAAAGGATTGTATCGTAGAAATTTTTTTTTTAATGATGCGGTTAATTTAGATTAGTTTGGCAGAGTAGTGCATCGACGTTAGAATTCGAATAT  
  
CG27Head\_lig-seq 2144 GTCTTAGGCAGCTGGTAGTTATTGTGGCAGATTAGTGCGAAGAAGTTAAGTTTCTTATATGAATTTATTCCATTAATAGTGTGAGATTTAGGTGTTTTTTTTGGGGAGATTATTATGTTG  
CG27Body\_lig-seq 2150 GTCTTAGGCAGCTGGTAGTTATTGTGGCAGATTAGTGCGAAGAAGTTAAGTTTCTTATATGAATTTATTCCATTAATAGTGTGAGATTTAGGTGTTTTTTTTGGGGAGATTATTATGTTG  
CG27Legs\_lig-seq 2138 GTCTTAGGCAGCTGGTAGTTATTGTGGCAGATTAGTGCGAAGAAGTTAAGTTTCTTATATGAATTTATTCCATTAATAGTGTGAGATTTAGGTGTTTTTTTTGGGGAGATTATTATGTTG  
  
CG27Head\_lig-seq 2264 GTTATAGTTTTGGTTGGAGTAGCTTTTGTTACTTTACTTGAGCGTAAGATTTTGGGTTATATTCAGCTTCGAAAGGGTCCTAATAGGGTGGGTTATATGGGTTTGTTACAGCCTATTGCT  
CG27Body\_lig-seq 2270 GTTATAGTTTTGGTTGGAGTAGCTTTTGTTACTTTACTTGAGCGTAAGATTTTGGGTTATATTCAGCTTCGAAAGGGTCCTAATAGGGTGGGTTATATGGGTTTGTTACAGCCTATTGCT  
CG27Legs\_lig-seq 2258 GTTATAGTTTTGGTTGGAGTAGCTTTTGTTACTTTACTTGAGCGTAAGATTTTGGGTTATATTCAGCTTCGAAAGGGTCCTAATAGGGTGGGTTATATGGGTTTGTTACAGCCTATTGCT  
  
CG27Head\_lig-seq 2384 GATGCTGTTAAGTTATTTAGAAGGGAAATTGTTTGATTGAAGTTAATAAGATTTTATTTTTATATTTATAGTCCCATTTTTGGATTAGCTTTTTTTTCTTTTTTTTTGTGGGTGGTTTTT  
CG27Body\_lig-seq 2390 GATGCTGTTAAGTTATTTAGAAGGGAAATTGTTTGATTGAAGTTAATAAGATTTTATTTTTATATTTATAGTCCCATTTTTGGATTAGCTTTTTTC----TTTTTTTGTGGGTGGTTTTT  
CG27Legs\_lig-seq 2378 GATGCTGTTAAGTTATTTAGAAGGGAAATTGTTTGATTGAAGTTAATAAGATTTTATTTTTATATTTATAGTCCCATTTTTGGATTAGCTTTTTTC---TTTTTTTTGTGGGTGGTTTTT  
  
CG27Head\_lig-seq 2504 CCTGTAGTTTTTGGGTCTTTTGATTTTATTTTGGGGTTGATGTATTTTTTTTGTTTTAGAAGATTGGGGGTATATGTTTTGTTTGGTTGTGGTTGGTCCTCAAATTCTGTTTATTCTTTA  
CG27Body\_lig-seq 2506 CCTGTAGTTTTTGGGTCTTTTGATTTTATTTTGGGGTTGATGTATTTTTTTTGTTTTAGAAGATTGGGGGTATATGTTTTGTTTGGTTGTGGTTGGTCCTCAAATTCTGTTTATTCTTTA  
CG27Legs\_lig-seq 2495 CCTGTAGTTTTTGGGTCTTTTGATTTTATTTTGGGGTTGATGTATTTTTTTTGTTTTAGAAGATTTGGGGTATATGTTTTGTTTGGTTGTGGTTGGTCCTCAAATTCTGTTTATTCTTTA  
  
CG27Head\_lig-seq 2624 TTGGGGGGCTATGCGTGGTGTGGCTCAGATAATTTCTTATGAGGTTAGATTGATTTTTATTGTCTTAAGATGTGTTATTTTAAGAAGCTCCTACGATTTTGAGGTAATTTCTGATTGACA  
CG27Body\_lig-seq 2626 TT-GGGGGCTATGCGTGGTGTGGCTCAGATAATTTCTTATGAGGTTAGATTGATTTTTATTGTCTTGAGATGTGTTATTTTAAGAAGCTCTTACGATTTTGAGGTAATTTCTGATTGACA  
CG27Legs\_lig-seq 2615 TT-GGGGGCTATGCGTGGTGTGGCTCAGATAATTTCTTATGAGGTTAGATTGATTTTTATTGTCTTGAGATGTGTTATTTTAAGAAGCTCTTACGATTTTGAGGTAATTTCTGATTGACA  
  
CG27Head\_lig-seq 2744 AAGCTTGTTTTGGTATTTTATTTTTC-TTTTTCCTTTAATGATTATTTGGGTAGTTTCTTGTTTAGCTGAGACTAATCGTACACCTTTTGATTTTGCTGAGGGGGGAGTCAGAGTTGGTT  
CG27Body\_lig-seq 2745 AAGCTTGTTTTGGTATTTTATTTTTCTTTTTTCCTTTAATGATTATTTGGGTAGTTTCTTGTTTAGCTGAGACTAATCGTACACCTTTTGATTTTGCTGA-GGGGGAGTCAGAGTTGGTT  
CG27Legs\_lig-seq 2734 AAGCTTGTTTTGGTATTTTATTTTTC-TTTTTCCTTTAATGATTATTTGGGTAGTTTCTTGTTTAGCTGAGACTAATCGTACACCTTTTGATTTTGCTGA-GGGGGAGTCAGAGTTGGTT  
  
CG27Head\_lig-seq 2863 TCTGGTTTTAATGTTGAGTATGGAGGATTTGGTTTTGCTTTTATTTTTATGGCTGAGTATGGTGTAATTATATTAATATCTTGTTTAGTAGTTGTCTTGTTTTTAGGTGGTGTTGATTGA  
CG27Body\_lig-seq 2864 TCTGGTTTTAATGTTGAGTATGGAGGATTTGGTTTTGCTTTTATTTTTATGGCTGAGTATGGTGTAATTATATTAATATCTTGTTTAGTAGTTGTCTTGTTTTTAGGTGGTGTTAATTGA  
CG27Legs\_lig-seq 2852 TCTGGTTTTAATGTTGAGTATGGAGGATTTGGTTTTGCTTTTATTTTTATGGCTGAGTATGGTGTAATTATATTAATATCTTGTTTAGTAGTTGTCTTGTTTTTAGGTGGTGTTAATTGA  
  
CG27Head\_lig-seq 2983 GTTGTGTTTTTGGGTGGTTTTATGGTTAGATTTTGGATTTGGGTGCGAGGGGCTTATCCGCGTTTTCGGTATGATAAATTAATAAGATTGGCTTGGCGAGGTTATTTGCCTGTGTCTGTA  
CG27Body\_lig-seq 2984 GTTGTGTTTTTGGGTGGTTTTATGGTTAGATTTTGGATTTGGGTGCGAGGAGCTTATCCGCGTTTTCGGTATGATAAATTAATAAGATTGGCTTGGCGAGGTTATTTGCCTGTGTCTGTA  
CG27Legs\_lig-seq 2972 GTTGTGTTTTTGGGTGGTTTTATGGTTAGATTTTGGATTTGGGTGCGAGGAGCCTATCCGCGTTTTCGGTATGATAAATTAATAAGATTGGCTTGGCGAGGTTATTTGCCTGTGTCTGTA  
  
CG27Head\_lig-seq 3103 AATTATTTAATTGGAAGATTTGGTTTTGTTTTATTTGTTTTTTTATTATCAGACAATAGTTGAATTAGAATAAATGCTTTGGGGGCGTTAGGTGGATAAATTTATCCTTGTTTGATTATA  
CG27Body\_lig-seq 3104 AATTATTTAATTGGAAGATTTGGTTTTGTTTTATTTGTTTTTTTATTATCAGACAATAGTTGAATTAGAATAAATGCTTTGGGGGCGTTAGGTGGATAAATTTATCCTTGTTTGATTATA  
CG27Legs\_lig-seq 3092 AATTATTTAATTGGAAGATTTGGTTTTGTTTTATTTGTTTTTTTATTATCAGACAATAGTTGAATTAGAATAAATGCTTTGGGGGCGTTAGGTGGAT---TTACCCCTTGTTTGATTATA  
  
CG27Head\_lig-seq 3223 TTATGGTAAGCTTGGGGGTTGTATTGTTAATAGGTGGTTTTGTTGGTTTATTAAGAAGCTATAGGCATGTTTTAAATATACTTCTTAGATTGGAGTTAGTTATGTTGTCACTGTTGTATT  
CG27Body\_lig-seq 3224 TTATGGTAAGCTTGGGGGTTGTATTGTTAATAGGTGGTTTTGTTGGTTTATTAAGAAGCTATAGGCATGTTTTAAATATACTTCTTAGATTGGAGTTAGTTATGTTGTCACTGTTGTATT  
CG27Legs\_lig-seq 3209 TTATGGTAAGCTTGGGGGTTGTATTGTTAATAGGTGGTTTTGTTGGTTTATTAAGAAGCTATAGGCATGTTTTAAATATACTTCTTAGATTGGAGTTAGTTATGTTGTCACTGTTGTATT  
  
CG27Head\_lig-seq 3343 TTTTGGGTTTATGGGAAATGATGAGTTTAAATGACTTGGTTTTTATGTTGTGTTTTATTGTATTTATGGTTGGTGAAGGAGTTTTAGGTTTGAGAATTTTGATTACTCTTGTTCGTGGTC  
CG27Body\_lig-seq 3344 TTTTGGGTTTATGGGAAATGATGAGTTTAAATGACTTGGTTTTTATGTTGTGTTTTATTGTATTTATGGTTGGTGAAGGAGTTTTAGGTTTGAGAATTTTGATTACTCTTGTTCGTGGTC  
CG27Legs\_lig-seq 3329 TTTTGGGTTTATGGGAAATGATGAGTTTAAATGACTTGGTTTTTATGTTGTGTTTTATTGTATTTATGGTTGGTGAAGGAGTTTTAGGTTTGAGAATTTTGATTACTCTTGTTCGTGGTC  
  
CG27Head\_lig-seq 3463 ATGGTGGGGATTATTATTTGAGTTTTAATAGTTTACAGTGTTAGAGGTAGTAGGTGGTTTGTTTGGGTTGATAGTGATACTTGTCTTCAAGGTTGGTTGGTTTTATATTTATTTAG--TT  
CG27Body\_lig-seq 3464 ATGGTGGGGATTATTATTTGAGTTTTAATAGTTTACAGTGTTAGAGGTAGTAGGTGGTTTGTTTGGGTTGATAGTGATACTTGTCTTCAAGGTTGGTTGGTTTTATATTTATTTAGTTTT  
CG27Legs\_lig-seq 3449 ATGGTGGGGATTATTATTTGAGTTTTAATAGTTTACAGTGTTAGAGGTAGTAGGTGGTTTGTTTGGGTTGATAGTGATACTTGTCTTCAAGGTTGGTTGGTTTTATATTTATTTAG--TT  
  
CG27Head\_lig-seq 3581 ATATTTTTTATGATTATTTTTATTATAAGGGATTTTTATTTTGGGTTAGAGGATTGGCATTTGTATATGGGTT--AATTTGGTGGGGATAGAATGAGTGTTGGTTTTGTTTTGTTAAGAT  
CG27Body\_lig-seq 3584 TTATTTTTTATGATTATTTTTATTATAAGGGATTTTTATTTTGGGTTAGAGGATTGGCATTTGTATATGGGTTTATATTTGGTGGGGATAGAATGAGTGTTGGTTTTGTTTTGTTAAGAT  
CG27Legs\_lig-seq 3567 TTAATTTTTATGATTATTTTTATTATAAGGGATTTTTATTTTGGGTTAGAGGATTGGCATTTGTATATGGGTT--TATTTGGTGGGGATAGAATGAGTGTTGGTTTTGTTTTGTTAAGAT  
  
CG27Head\_lig-seq 3699 TTTGGGTTATTTTATTAATAATTTTATCTAGTTGTGTTAGGTATAATTCTAAGGGAGCTATTTTTCTTTATGTTACTATGGTTTTTATTTTGATAATGGTTTTTATTAGTTTGGATTATA  
CG27Body\_lig-seq 3704 TTTGAGTTATTTTATTAATAATTTTATCTAGTTGTGTTAGGTATAATTCTAAGGGAGCTATTTTTCTTTATGTTACTATGGTTTTTATTTTGATAATGGTTTTTATTAGTTTGGATTATA  
CG27Legs\_lig-seq 3685 TTTGAGTTATTTTATTAATAATTTTATCTAGTTGTGTTAGGTATAATTCTAAGGGAGCTATTTTTCTTTATGTTACTATGGTTTTTATTTTGATAATGGTTTTTATTAGTTTGGATTATA  
  
CG27Head\_lig-seq 3819 TAATGTTTTACTTAATGTTTGAGGCTATTTTAATTCCTACTGTAATTTTGATTTTGGGTTGGGGGTATCAGCCAGAGCGTTTGAGAGCTGGGATTTATTTGTTGTTTTATACTGTTTTTT  
CG27Body\_lig-seq 3824 TAATGTTTTACTTAATGTTTGAGGCTATTTTAATTCCTACTGTAATTTTGATTTTGGGTTGGGGGTATCAGCCAGAGCGTTTGAGAGCTGGGATTTATTTGTTGTTTTATACTGTTTTTT  
CG27Legs\_lig-seq 3805 TAATGTTTTACTTAATGTTTGAGGCTATTTTAATTCCTACTGTAATTTTGATTTTGGGTTGGGGGTATCAGCCAGAGCGTTTGAGAGCTGGGATTTATTTGTTGTTTTATACTGTTTTTT  
  
CG27Head\_lig-seq 3939 GTTCTATGCCTTTACTATTACTGATTGTTTTGGTTAAGATGGTATTGGGAGGTATGGGAAGATTGGAGAGTGCTTCTTTTGGCTTAGGTTGACTTCCTAGTAGTCTTTATTTGCTTTGTT  
CG27Body\_lig-seq 3944 GTTCTATGCCTTTACTATTACTGATTGTTTTGGTTAAGATGGTATTGGGAGGTATGGGAAGATTGGAGAGTGCTTCTTTTGGCTTAGGTTGACTTCCTAGTAGTCTTTATTTGCTTTGTT  
CG27Legs\_lig-seq 3925 GTTCTATGCCTTTACTATTACTGATTGTTTTGGTTAAGATGGTATTGGGAGGTATGGGAAGATTGGAGAGTGCTTCTTTTGGCTTAGGTTTACTTCCTAGTAGTCTTTATTTGCTTTGTT  
  
CG27Head\_lig-seq 4059 TTGGGAGAATAATGGCTTTTTTAGTAAGGGTTCCTTTTGTTTGGTTTACATCTTTGAATACCAAAGGCTCATGTTGAAGCCCCTGTTTCTGGTTCTATGATTTTGGCTGGTGTTTTATTG  
CG27Body\_lig-seq 4064 TTGGGAGAATAATGGCTTTTTTAGTAAGGGTTCC-TTTGTTTGGTTTACATCTTTGATTACCAAAGGCTCATGTTGAAGCCCCTGTTTCTGGTTCTATGATTTTGGCTGGTGTTTTATTG  
CG27Legs\_lig-seq 4045 TTGGGAGAATAATGGCTTTTTTAGTAAGGGTTCC-TTTGTTTGGTTTACATCTTTGATTACCAAAGGCTCATGTTGAAGCCCCTGTTTCTGGTTCTATGATTTTGGCTGGTGTTTTATTG  
  
CG27Head\_lig-seq 4179 AAGCTGGGGGGTTATGGATTAATGCGTTTTAGATTTTATGGTTGGATTGTTTTTAAGGATTTTGGTTTTTTTTTAATTAGAGTTTCTTTATTAGGTGGAATTGGTTTGAGATTGGTGTGT  
CG27Body\_lig-seq 4183 AAGCTGGGGGGCTATGGATTAATGCGTTTTAGATTTTATGGTTGGATTGTTTTTAAGGATTTTGGTTTTTTTTTAATTAGAGTTTCTTTATTAGGTGGAATTGGTTTGAGATTGGTGTGT  
CG27Legs\_lig-seq 4164 AAGCTGGGGGGCTATGGATTAATGCGTTTTAGATTTTATGGTTGGATTGTTTTTAAGGATTTTGGTTTTTTTTTAATTAGAGTTTCTTTATTAGGTGGAATTGGTTTGAGATTGGTGTGT  
  
CG27Head\_lig-seq 4299 TTACGTCAAGTGGATTTAAGGGCTTTGATTGCTTATTCATCTGTTGTTCATATGGGGTTAGTAGT-AGGGGTATTATATCGGGTTTTATAGTTGGGTGAGTTGGGGCTTTTGTTATGATG  
CG27Body\_lig-seq 4303 TTACGTCAAGTGGATTTAAGGGCTTTGATTGCTTATTCATCTGTTGTTCATATGGGGTTAGTAGT-GGGGGTATTATATCGGGTTTTATAGTTGGGTGAGTTGGGGCTTTTGTTATGATG  
CG27Legs\_lig-seq 4284 TTACGTCAAGTGGATTTAAGGGCTTTGATTGCTTATTCATCTGTTGTTCATATGGGGTTAGTAGTAGGGGGTATTATATCTGGTTTTATAGTTGGGTGAGTTGGGGCTTTTGTTATGATG  
  
CG27Head\_lig-seq 4418 GTTGGACATGGTTTATGTTCTTCTGGTTTATTTTATTATGCGGGTATTAATTATGATCGTCTTGGTAGTCGTAGAGTTTTAATGAACAGGGGTTTAATATTGGTTTTTCCTAGAAGAGTA  
CG27Body\_lig-seq 4422 GTTGGACATGGTTTATGTTCTTCTGGTTTATTTTATTATGCGGGTATTAATTATGATCGTCTTGGTAGTCGTAGAGTTTTAATGAACAGGGGTTTAATATTGGTTTTTCCTAGAAGAGTA  
CG27Legs\_lig-seq 4404 GTTGGACATGGTTTATGTTCTTCTGGTTTATTTTATTATGCGGGTATTAATTATGATCGTCTTGGTAGTCGTAGAGTTTTAATGAACAGGGGTTTAATATTGGTTTTTCCTAGAAGAGTA  
  
CG27Head\_lig-seq 4538 CTATTTTGGTTTTTATTTGTAAGATCAAATATAGCGGCTCCACCCTCTTTGAATTTGCTTGGTGAGTTAATATTGCTTGGTGGGGTATTGGCAGTTAGATCTTGACTTATAATTTTATTA  
CG27Body\_lig-seq 4542 CTATTTTGGTTTTTATTTGTAAGATCAAATATAGCGGCTCCACCCTCTTTGAATTTGCTTGGTGAGTTAATATTGCTTGGTGGGGTATTGGCAGTTAGATCTTGACTTATAATTTTATTA  
CG27Legs\_lig-seq 4524 CTATTTTGGTTTTTATTTGTAAGATCAAATATAGCGGCCCCACCCTCTTTGAATTTGCTTGGTGAGTTAATATTGCTTGGTGGGGTATTGGCAGTTAGATCTTGACTTATAATTTTATTA  
  
CG27Head\_lig-seq 4658 TTATTAAGCTCTTTT-TTTAGCGGTGTGTATTGTTTGTATATATTTGGTTGAGTTCAGCATGGAGATGCTAGAAGGTTTATTCGTGGATCTGATGGGATGATTATGATTGAAGGTTTAAT  
CG27Body\_lig-seq 4662 TTATTAAGCTCTTTTCCTTAGCGGTGTGTATTGTTTGTATATATTTGGTTGAGTTCAGCATGGAGATGCTAGAAGGTTTATTCGTGGATCTGATGGGATGATTATGATTGAAGGTTTAAT  
CG27Legs\_lig-seq 4644 TTATTAAGCTCTTTT-TTTAGCGGTGTGTATTGTTTGTATATATTTGGTTGAGTTCAGCATGGAGATGCTAGAAGGTTTATTCGTGGATCTGATGGGATGATTATGATTGAAGGTTTAAT  
  
CG27Head\_lig-seq 4777 TTCTTTACTACATTGATTACCATTAAATTGTTTGTTTTTAAAAGGGGGATTTATTTTTATTTTAGTTCGTGTAGTTTAAGAAAAATTATGGAATGTGGTTCCTTAGATAACCCAGGTTCG  
CG27Body\_lig-seq 4782 TTCTTTACTACATTGATTACCATTAAATTGTTTGTTTTT-AAAGGGGGATTTATTTTTATTTTAGTTCGTGTAGTTTAAGAAAAATTATGGAATGTGGTTCCTTAGATAACCCAGGTTCG  
CG27Legs\_lig-seq 4763 TTCTTTACTACATTGATTACCATTAAATTGTTTGTTTTT-AAAGGGGGATTTATTTTTATTTTAGTTCGTGTAGTTTAAGAAAAATTATGGAATGTGGTTCCTTAGATAACCCAGGTTCG  
  
CG27Head\_lig-seq 4897 CGGACAAGATGTTTTGGTTATATTTTGTTATTTTTATGGGCCTTTTTTTGTTGAGATTATTGGAGTTTTTGTTTTGGGGTGTTGTCTTTATATGGTGGATGAGTAATTATTATGGAGTGG  
CG27Body\_lig-seq 4901 CGGACAAGATGTTTTGGTTATATTTTGTTATTTTTATGGGCCTTTTTTTGTTGAGATTATT-GAGTTTTTGTTTTGGGGTGTTGTCTTTATATGGTGGATGAGTAATTATTATGGAGTGG  
CG27Legs\_lig-seq 4882 CGGACAAGATGTTTTGGTTATATTTTGTTATTTTTATGGGCCTTTTTTTGTTGAGATTATT-GAGTTTTTGTTTTGGGGTGTTGTCTTTATATGGTGGATGAGTAATTATTATGGAGTGG  
  
CG27Head\_lig-seq 5017 GTTATTATTCGTGTTTTTGGGTTGGATTTAGGTTTTGTTTTAATGTTTGATTGGATGAGATTAGTTTTTATAGGTGTAGTTCTTTTTAATTTCAAGTTGTGTATTTTATTATAGTGTGGG  
CG27Body\_lig-seq 5020 GTTATTATTCGTGTTTTA-GGTTGGATTTAGGTTTTGTTTTAATGTTTGATTGGATGAGATTAGTTTTTATAGGTGTAGTTC-TTTTAATTTCAAGTTGTGTATTTTATTATAGTGTGGG  
CG27Legs\_lig-seq 5001 GTTATTATTCGTGTTTTAGGGTTGGATTTAGGTTTTGTTTTAATGTTTGATTGGATGAGATTAGTTTTTATAGGTGTAGTTC-TTTTAATTTCAAGTTGTGTATTTTATTATAGTGTGGG  
  
CG27Head\_lig-seq 5137 TTATATGGGGGGGGATAAGAATATTTTACGGTTTGGTTTATTGGTTTTATTATTTGTTATTTCTATGTTGTTTGTTATTGTTTCTCCTAGATTTATTAGAATTTTGCTTGGATGAGATGG  
CG27Body\_lig-seq 5138 TTATATAGAGGGGGATAAGAATATTTTACGGTTTGGTTTATTGGTTTTATTATTTGTTATTTCTATGTTGTTTGTTATTGTTTCTCCTAGATTTATTAGAATTTTGCTTGGATGAGATGG  
CG27Legs\_lig-seq 5120 TTATATAGAGGGGGATAAGAATATTTTACGGTTTGGTTTATTGGTTTTATTATTTGTTATTTCTATGTTGTTTGTTATTGTTTCTCCTAGATTTATTAGAATTTTGCTTGGATGAGATGG  
  
CG27Head\_lig-seq 5257 TTTGGGGTTAGTTTCTTATTGTTTAGTCATTTATTATCAGAATTATCGTTCTTATAATGCTGGTATAATTACTGGTGTAACTAATCGTCTTGGGGATGTGGGTCTTTTGTTAACAATTGG  
CG27Body\_lig-seq 5258 TTTGGGGTTAGTTTCTTATTGTTTAGTCATTTATTATCAGAATTATCGTTCTTATAATGCTGGTATAATTACTGGTGTAACTAATCGTCTTGGGGATGTGGGTCTTTTGTTAACAATTGG  
CG27Legs\_lig-seq 5240 TTTGGGGTTAGTTTCTTATTGTTTAGTCATTTATTATCAGAATTATCGTTCTTATAATGCTGGTATAATTACTGGTGTAACTAATCGTCTTGGGGATGTTGGTCTTTTGTTAACAATTGG  
  
CG27Head\_lig-seq 5377 TTTGTTGTTTAGATATGGGAGATGGAATTTTTATTCTTTTGATATTTCTGGTGGGATGTGGGGTATTATTTCTATTTTGGTTTTATTGGCTGGTTTTACTAAAAGAGCTCAGATTCCATT  
CG27Body\_lig-seq 5378 TTTGTTGTTTAGATATGGGAGATGGAATTTTTATTCTTTTGATATTTCTGGTGGGATGTGGGGTATTATTTCTATTTTGGTTTTATTGGCTGGTTTTACTAAAAGAGCTCAGATTCCATT  
CG27Legs\_lig-seq 5360 TTTGTTGTTTAGATATGGGAGATGGAATTTTTATTCTTTTGATATTTCTGGTGGGATGTGGGGTATTATTTCTATTTTGGTTTTATTGGCTGGTTTTACTAAAAGAGCTCAGATTCCATT  
  
CG27Head\_lig-seq 5497 TTCTGCTTGATTACCAGCTGCAATAGCAGCTCCAACACCTGTTTCGGCGTTGGTTCATTCTTCTACTTTGGTTACGGCTGGGGTTTATTTGTTAATTCGTTTTTATGGGGTATTAGGTC-  
CG27Body\_lig-seq 5498 TTCTGCTTGATTACCAGCTGCAATAGCAGCTCCAACACCTGTTTCGGCGTTGGTTCATTCTTCTACTTTGGTTACGGCTGGGGTTTATTTGTTAATTCGTTTTTATGGGGTATTAGGTCT  
CG27Legs\_lig-seq 5480 TTCTGCTTGATTACCAGCTGCAATAGCAGCTCCAACACCTGTTTCGGCGTTGGTTCATTCTTCTACTTTGGTTACGGCTGGGGTTTATTTGTTAATTCGTTTTTATGGGGTATTAGGTCT  
  
CG27Head\_lig-seq 5616 TTTTTGGTGGGTGGTTATAGTAATTGTGTATGGGGGGGTGTTAACTATGTTTATAGCTGGGATGGTGGCTAATTTTGAAATGGATATGAAGAAGATTATTGCTTTGTCAACTCTTAGACA  
CG27Body\_lig-seq 5618 TTTTTGGTGGGTGGTTATAGTAATTGTGTAT-GGGGGGTGTTAACTATGTTTATAGCTGGGATGGTGGCTAATTTTGAAATGGATATGAAGAAGATTATTGCTTTGTCAACTCTTAGACA  
CG27Legs\_lig-seq 5600 TTTTTGGTGGGTGGTTATAGTAATTGTGTATGGGGGGGTGTTAACTATGTTTATAGCTGGGATGGTGGCTAATTTTGAAATGGATATGAAGAAGATTATTGCTTTGTCAACTCTTAGACA  
  
CG27Head\_lig-seq 5736 GTTGGGGTTGATAATGATATCTGTGGGTCCTTGGTAATGTTTATTTAGCTTTTTTTTCATTTGGTGGTTCACGCTATTTTTAAGGCCTTATTATTTTTATGTGGTGGTAAGATAATTCAC  
CG27Body\_lig-seq 5737 GTTGGGGTTGATAATGATATCTGTGGGT-CTTGGTAATGTTTATTTAGCTTTTTTTTCATTTGGTGGTTCACGCTATTTTTAAGGCCTTATTATTTTTATGTGGTGGTAAGATAATTCAC  
CG27Legs\_lig-seq 5720 GTTGGGGTTGATAATGATATCTGTGGGT-CTTGGTAATGTTTATTTAGC-TTTTTTTCATTTGGTGGTTCACGCTATTTTTAAGGCCTTATTATTTTTATGTGGTGGTAAGATAATTCAC  
  
CG27Head\_lig-seq 5856 AGCTTTGGGGGTGAACAAGATTTACGTCATATGGGTGGGATTGTTGTTGGTTTGCCTATTAGATCTGTTAGTTTAAATGTGGCCAATTTTTCTTTATGTGGGATGCCTTTTATATCTGGT  
CG27Body\_lig-seq 5856 AGCTTTGGGGGTGAACAAGATTTACGTCATATGGGTGGGATTGTTGTTGGTTTGCCTATTAGATCTGTTAGTTTAAATGTGGCCAATTTTTCTTTATGTGGGATGCCTTTTATATCTGGT  
CG27Legs\_lig-seq 5838 AGCTTTGGGGGTGAACAAGATTTACGTCATATGGGTGGGATTGTTGTTGGTTTGCCTATTAGATCTGTTAGTTTAAATGTGGCCAATTTTTCTTTATGTGGGATGCCTTTTATATCTGGT  
  
CG27Head\_lig-seq 5976 TTTTATTCTAAGGATCTTATTTTGGAGATAAGAGGTTCTTGGAGAATAAATTTGATTTTGTTTTTTTTAGTTGTTATTGGTACTATTTTTACTTTCATATACACTATTAAATTCTGTATG  
CG27Body\_lig-seq 5976 TTTTATTCTAAGGATCTTATTTTGGAGATAAGAGGTTCTTGGAGAATAAATTTGATTTTG-TTTTTTTAGTTGTTATTGGTACTATTTTTACTTTCATATACACTATTAAATTCTGTATG  
CG27Legs\_lig-seq 5958 TTTTATTCTAAGGATCTTATTTTGGAGATAAGAGGTTCTTGGAGAATAAATTTGATTTTGTTTTTTTTAGTTGTTATTGGTACTATTTTTACTTTCATATACACTATTAAATTCTGTATG  
  
CG27Head\_lig-seq 6096 AGAAGAATAATTGTGTACTATGGTGGTTGGGGTTATAGATGGGACGATTCTGATTTTAATTATATTTTTCCTATATTGGTCTTATCTATTGGCGGAGTTGTAGGAGGGTCTTTTTTATTT  
CG27Body\_lig-seq 6095 AGAAGAATAATTGTGTACTATGGTGGTTGGGGTTATAGATGGGACGATTCTGATTTTAATTATATTTTTCCTATATTGGTCTTATCTATTGGCGGAGTTGTAGGAGGGTCTTTTTTATTT  
CG27Legs\_lig-seq 6078 AGAAGAATAATTGTGTACTATGGTGGTTGGGGTTATAGATGGGACGATTCTGATTTTAATTATATTTTTCCTATATTTGTCTTATCTATTGGCGGAGTTGTAGGAGGGTCTTTTTTATTT  
  
CG27Head\_lig-seq 6216 TGGTTGGGGGTGCCTGGTTATGATATAAATTTTGTTTATGGAGTTGATAAGATTTGGCCATCTTTTTGTAGTTTTTTTGGTTTTTTTTTGTTGGGTTATTACCTAGATTTAGTTCTGGGG  
CG27Body\_lig-seq 6215 TGGTTGGGGGTGCCTGGTTATGATATAAATTTTGTTTATGGAGTTGATAAGATTTGGCCATCTTTTTTAGTGTTTTTGGTTTGTTTTTTGTTGGGTTATTA-CTAGATTTAGTGCTGGGG  
CG27Legs\_lig-seq 6198 TGGTTGGGGGTGCCTGGTTATGATATAAATTTTGTTTATGGAGTTGATAAGATTTGGCCATCTTTTTTAGTGTTTTTGGTTTTTTTTTTGTTGGGTTATTA-CTAGATTT-GTGCTGGGG  
  
CG27Head\_lig-seq 6336 AA--GATTAGTTTGGGTTGGGTTTAAGAGATTTTTTTGGGGGTATATGGTTTATTTCTGGATTAAGTACTGGTTGTATTTATTATCCTCTTGATATAGGTCGTTTAGTGATTAATTACTT  
CG27Body\_lig-seq 6334 GATTAATTAGTTTGGG-----TTTAAGAGATTTTTTTGGAGGTATATGGTTTATTTCTGGATTAAGTACTGGTTGTATTTATTATCCTCTTGATATAGGTCGTTTAGTGATTAATTACTT  
CG27Legs\_lig-seq 6316 G---AATTAGTTTGGGT----TTTAAGAGATTTTTTTGGAGGTATATGGTTTATTTCTGGATTAAGTACTGGTTGTATTTATTATCCTCTTGATATAGGTCGTTTAGTGATTAATTACTT  
  
CG27Head\_lig-seq 6454 TGATGGCGGGTGGGGAAAAG-GATTGGTGGTCAGGGTTTATATAGATTTGAGAAGGGTGTTGGTTCTTTGTTGCAGGCCTTCCAAGGGGTTGTTTTTACGGTTGTTATGTTTATTCTTAT  
CG27Body\_lig-seq 6449 TGATGGCGGGTGGGGGGAAATGATTGGTGGTCAGGGTTTATATAGATTTGAGAAGGGTGTTGGTTCTTTGTTGCAGGCCTTCCAAGGGGTTGTTTTTACGGTTGTTATGTTTATTCTTAT  
CG27Legs\_lig-seq 6429 TGATGGCGGGTGGGGAGAAGTTATTGGTGGTCAGGGTTTATATAGATTTGAGAAGGGTGTTGGTTCTTTGTTGCAGGCCTTCCAAGGGGTTGTTTTTACGGTTGTTATGTTTATTCTTAT  
  
CG27Head\_lig-seq 6573 TATGTGGGTAATAATTGCTTGATTTTCTATAATTTAGTTAAGGTAGCTTAAGATTGAGCGTAGCTCTGAAGTTGCTAAGGTGATTTATTGTCCTTTAATGGAAAATGAGATAGTTTAAGT  
CG27Body\_lig-seq 6569 TATGTGGGTAATAATTGCTTGATTTTCTATAATTTAGTTAAGGTAGCTTAAGATTGAGCGTAGCTCTGAAGTTGCTAAGGTGATTTATTGTCCTTTAATGGAAAATGAGATAGTTTAAGT  
CG27Legs\_lig-seq 6549 TATGTGGGTAATAATTGCTTGATTTTCTATAATTTAGTTAAGGTAGCTTAAGATTGAGCGTAGCTCTGAAGTTGCTAAGGTGATTTATTGTCCTTTAATGGAAAATGAGATAGTTTAAGT  
  
CG27Head\_lig-seq 6693 TTGAACGGTAAATTGTAAATTTATTGGTGGTTGTAACCTCTTATTATTTACGCTGGGGTGTTAGAGCACGATAAATCTTGATTTTATATGTTTATAGGGAATATAACTGTGTGGGCAAAG  
CG27Body\_lig-seq 6689 TTGAACGGTAAATTGTAAATTTATTGGTGG-TGTAACCTCTTATTATTTACGCTGGGGTGTTAGAGCACGATAAATCTTGATTTTATATGTTTATAGGG-ATATAACTGTGTGGGCAAAG  
CG27Legs\_lig-seq 6669 TTGAACGGTAAATTGTAAATTTATTGGTGG-TGTAACCTCTTATTATTTACGCTGGGGTGTTAGAGCACGATAAATCTTGATTTTATATGTTTATAGGG-ATATAACTGTGTGGGCAAAG  
  
CG27Head\_lig-seq 6813 TAGTTTATTAGAATAATAAATTGCAAGTTTATAGGAGAGTTACTTCTTGTCCTAGTTTGGTGTCTGATAAAAGAGTTATTGTGATACGATAGAGAATGCTATTTAGCCTGAGCTGAGAAA  
CG27Body\_lig-seq 6807 TAGTTTATTAGAATAATAAATTGCAAGTTTATAGGAGAGTTACTTCTTGTCCTAGTTTGGTGTCTGATAAAAGAGTTATTGTGATACGATAGAGAATGCTATTTAGCCTGAACTGAGAAA  
CG27Legs\_lig-seq 6787 TAGTTTATTAGAATAATAAATTGCAAGTTTATAGGAGAGTTACTTCTTGTCCTAGTTTGGTGTCTGATAAAAGAGTTATTGTGATACGATAGAGAATGCTATTTAGCCTGAACTGAGAAA  
  
CG27Head\_lig-seq 6933 AGATAAGCTAAGAAAGCTGATGGGTTCATATCTCATAAATAGATTAATTCTTCTTTTTATTGTGGGTAGTTTTAAGGTTTATTTATTTGAGATTTTTGATTGGGGGGAACTTTAATTGCT  
CG27Body\_lig-seq 6927 AGATAAGCTAAGAAAGCTGATGGGTTCATATCTCATAAATAGATTAATTCTTCTTTTTATTGTGGGTAGTTTTAAGGTTTATTTATTTGAGATTTTTGATT-GGGGGAACTTTAATTGCT  
CG27Legs\_lig-seq 6907 AGATAAGCTAAGAAAGCTGATGGGTTCATATCTCATAAATAGATTAATTCTTCTTTTTATTGTGGGTAGTTTTAAGGTTTATTTATTTGAGATTTTTGATT--GGGGAACTTTAATTGCT  
  
CG27Head\_lig-seq 7053 GTAACCGGCTCTTCTTGGTTTGTGGTTTGGGTTGGGCTAGAGATGAATATAATATCCTTTGTTCCCTTAGTTAACTTGAGGAGTAAGATTAATTCTGAGGCTTTGTTTAAATATTTTTTA  
CG27Body\_lig-seq 7046 GTAACCGGCTCTTCTTGGTTTGTGGTTTGGGTTGGGCTAGAGATGAATATAATATCCTTTGTTCCCTTAGTTAACTTGAGGAGTAAGATTAATTCTGAGGCTTTGTTTAAATATTTTTTA  
CG27Legs\_lig-seq 7025 GTAACCGGCTCTTCTTGGTTTGTGGTTTGGGTTGGGCTAGAGATGAATATAATATCCTTTGTTCCCTTAGTTAACTTGAGGAGTAAGATTAATTCTGAGGCTTTGTTTAAATATTTTTTA  
  
CG27Head\_lig-seq 7173 GTACAAGTTATTGGTTCTTTGTTATTGTTTTATGTAGGTGTTATTGGTTCTATTTGGATTAGATATGGTAATTTTATGTATGATATGTTTTCTAGAGGTGGGGTTTTAAGTAATATTGAT  
CG27Body\_lig-seq 7166 GTACAAGTTATTGGTTCTTTGTTATTGTTTTATGTAGGTGTTATTGGTTCTATTTGGATTAGATATGGTAATTTTATGTATGATATGTTTTCTAGAGGTGGGGTTTTATAAAATATTGAT  
CG27Legs\_lig-seq 7145 GTACAAGTTATTGGTTCTTTGTTATTGTTTTATGTAGGTGTTATTGGTTCTATTTGGATTAGATATGGTAATTTTATGTATGATATGTTTTCTAGAGGTGGGGTTTTA-GTAATATTGAT  
  
CG27Head\_lig-seq 7293 GGCTTTAAAGTTAGGAGCTAGCCCTGTTCATTTTTGGTTTCCATCTGTTGTTGAGGGGTTGGATTGGTTTGGAGTTTTATTATTGATGACTTGACAGAAGGTTGCCCCTCTTAGAGTTAT  
CG27Body\_lig-seq 7286 GGCTTTAAAGTTAGGAGCTAGCCCTGTTCATTTTTGGTTTCCATCTGTTGTTGAGGGGTTGGATTGGTTTGGAGTTTTATTATTGATGACTTGACAGAAGGTTGCCCCTCTTAGAGTTAT  
CG27Legs\_lig-seq 7264 GGCTTTAAAGTTAGGAGCTAGCCCTGTTCATTTTTGGTTTCCATCTGTTGTTGAGGGGTTGGATTGGTTTGGAGTTTTATTATTGATGACTTGACAGAAGGTTGCCCCTCTTAGAGTTAT  
  
CG27Head\_lig-seq 7413 TAGACTGGTAGATAGAATTTCTAGTTTGATTTTATTAGTTGGGGTTTTAAGTGTAATTGTTGGGGGTTTTGGGGGCTTAAATCAGCTATTATTGCGCGTAAGCTAATAGCTTATTCTTCT  
CG27Body\_lig-seq 7406 TAGACTGGTAGATAGAATTTCTAGTTTGATTTTATTAGTTGGGGTTTTAAGTGTAATTGTTGGGGGTTTTGGGGGCTTAAATCAGCTATTATT--GCGTAAGCTAATAGCTTATTCTTCT  
CG27Legs\_lig-seq 7384 TAGACTGGTAGATAGAATTTCTAGTTTGATTTTATTAGTTGGGGTTTTAAGTGTAATTGTTGGGGGTTTTGGGGGCTTAAATCAGCTATTATT--GCGTAAGCTAATAGCTTATTCTTCT  
  
CG27Head\_lig-seq 7533 ATTTCCCATTTAGGTTGACTTTGTTTAATTGTAATGATATCAGAGTGGGTTGGATTAGTTTATTTTATTAGTTATGTTTTAGTAAGAGGAGCTGTTATTATGTGTTTTATATTTAGAAGT  
CG27Body\_lig-seq 7524 ATTTCCCATTTAGGTTGACTTTGTTTAATTGTAATGATATCAGAGTGGGTTGGATTAGTTTATTTTATTAGTTATGTTTTAGTAAGAGGAGCTGTTATTATGTGTTTTATATTTAGAAGT  
CG27Legs\_lig-seq 7502 ATTTCCCATTTAGGTTGACTTTGTTTAATTGTAATGATATCAGAGTGGGTTGGATTAGTTTATTTTATTAGTTATGTTTTAGTAAGAGGAGCTGTTATTATGTGTTTTATATTTAGAAGT  
  
CG27Head\_lig-seq 7653 TTTATTCATGTTGGGCAGCTGTATTCGACTGGTTATGGGGTAGTTGGTTTTTTGGCCGTTTTTTATTTGGGGTTGTTTTCTTTAGGTGGTTTACCTCCTCTTTTTGGGTTTTTCCCAAAG  
CG27Body\_lig-seq 7644 TTTATTCATGTTGGGCAGCTGTATTCGACTGGTTATGGGGTAGTTGGTTTTTTGGCCGTTTTTTATTTGGGGTTGTTTTCTTTAGGTGGTTTACCTCCTCTTTTTGGGTTTTTCCCAAAG  
CG27Legs\_lig-seq 7622 TTTATTCATGTTGGGCAGCTGTATTCGACTGGTTATGGGGTAGTTGGTTTTTTGGCCGTTTTTTATTTGGGGTTGTTTTCTTTAGGTGGTTTACCTCCTCTTTTTGGGTTTTTCCCAAAG  
  
CG27Head\_lig-seq 7773 TGGTTAGGAATTATTTTATTGTTGGGGTCGGGTTATTTGTGGGTAGTTATATTTTTTGTTGTTGTGGGTTTATTGACTTTATATTTTTATTTACGGATGGGTTATGTGGGTTTAGTTGGT  
CG27Body\_lig-seq 7764 TGGTTAGGAATTATTTTATTGTTGGGGTCGGGTTATTTGTGGGTAGTTATATTTTTTGTTGTTGTGGGTTTATTGACTTTATATTTTTATTTACGGATGGGTTATGTGGGTTTAGTTGGT  
CG27Legs\_lig-seq 7742 TGGTTAGGAATTATTTTATTGTTGGGGT-GGGTTATTTGTGGGTAGTTATATTTTTTGTTGTTGTGGGTTTATTGACTTTATATTTTTATTTACGGATGGGTTATGTGGGTTTAGTTGGT  
  
CG27Head\_lig-seq 7893 TACAGAGGGTTTAACTTGTGGTCTTTTTATAGTTTTAAGGTG-CCTGCTTTCCTCTTCTTTTTTTTTCTGTGTTACTTATTTTCTTTTGCCATTTTCTGTATTTAGATTGTTATAAGATT  
CG27Body\_lig-seq 7884 TACAGAGGGTTTAACTTGTGGTCTTTTTATAGTTTTAAGGTGGCCTGCTTACTTCTTCTTTTTTCTT-TCTGTTACTTATTTTCTTTTGCCATTTTCTGTATTTAGATTGTTATAAGATT  
CG27Legs\_lig-seq 7861 TACAGAGGGTTTAACTTGTGGTCTTTTTATAGTTTTAAGGTG--CTGCTTACTTCTTCTTTTTTTTTCTCTGTTACTTATTTTCTTTTGCCATTTTCTGTATTTAGATTGTTATAAGATT  
  
CG27Head\_lig-seq 8012 TTAAGTTATTAAGACTGTTAATTTTCAAAGTTAGAAGTGATTTTTCAAATCTTGAATGCGGTGGTTGTATTCTACTAATCATAAGGATATTGGTACTATGTATTTAGTTTTTGGGGCTTG  
CG27Body\_lig-seq 8003 TTAAGTTATTAAGACTGTTAATTTTCAAAGTTAGAAGTGATTTTTCAAATCTTGAATGCGGTGGTTGTATTCTACTAATCATAAGGATATTGGTACTATGTATTTAGTTTTTGGGGCTTG  
CG27Legs\_lig-seq 7979 TTAAGTTATTAAGACTGTTAATTTTCAAAGTTAGAAGTGATTTTTCAAATCTTGAATGCGGTGGTTGTATTCTACTAATCATAAGGATATTGGTACTATGTATTTAGTTTTTGGGGCTTG  
  
CG27Head\_lig-seq 8132 GGCTGGGATTGTTGGTTCTGCTTTAAGAAGAATGATTCGTTTGGAATTGGGTCATTCTGGTAGTTTGATTGGGGATGATCAGATTTATAATGTAATTGTGACAGCCCATGCTTTTGTAAT  
CG27Body\_lig-seq 8123 GGCTGGGATTGTTGGTTCTGCTTTAAGAAGAATGATTCGTTTGGAATTGGGTCATTCTGGTAGTTTGATTGGGGATGATCAGATTTATAATGTAATTGTGACAGCCCATGCTTTTGTAAT  
CG27Legs\_lig-seq 8099 GGCTGGGATTGTTGGTTCTGCTTTAAGAAGAATGATTCGTTTGGAATTGGGTCATTCTGGTAGTTTGATTGGGGATGATCAGATTTATAATGTAATTGTGACAGCCCATGCTTTTGTAAT  
  
CG27Head\_lig-seq 8252 AATTTTTTTTATGGTTATGCCTATTATAATTGGGGGTTTTGGGAATTGATTGGTTCCTATTATGATTGGGGCTCCTGACATGGCTTTTCCTCGGATGAATAATTTAAGATTTTGGTTGTT  
CG27Body\_lig-seq 8243 AATTTTTTTTATGGTTATGCCTATTATAATTGGGGGTTTTGGGAATTGATTGGTTCCTATTATGATTGGGGCTCCTGACATGGCTTTTCCTCGGATGAATAATTTAAGATTTTGGTTGTT  
CG27Legs\_lig-seq 8219 AATTTTTTTTATGGTTATGCCTATTATAATTGGGGGTTTTGGGAATTGATTGGTTCCTATTATGATTGGGGCTCCTGACATGGCTTTTCCTCGGATGAATAATTTAAGATTTTGGTTGTT  
  
CG27Head\_lig-seq 8372 GCCCCCTTCTTTTTTTTTATTATTGGCTTCTTCTTTAGTAGAGAGGGGTGTGGGGACAGGGTGGACTGTTTATCCGCCTTTAGCCGCTAGTTTATTTCATGGGGGGCCAGCGGTTGATTT  
CG27Body\_lig-seq 8363 GCCCCCTTCTTTTTTTTTATTATTGGCTTCTTCTTTAGTAGAGAGGGGTGTGGGGACAGGGTGGACTGTTTATCCGCCTTTAGCCGCTAGTTTATTTCAT-GGGGGCCAGCGGTTGATTT  
CG27Legs\_lig-seq 8339 GCCCCCTTCTTTTTTTTTATTATTGGCTTCTTCTTTAGTAGAGAGGGGTGTGGGGACAGGGTGGACTGTTTATCCGCCTTTAGCCGCTAGTTTATTTCATGGGGGGCCAGCGGTTGATTT  
  
CG27Head\_lig-seq 8492 AGCTATTTTTTCTCTTCATTTGGCTGGGGCTTCTTCGATTTTAGGGGCAATTAATTTTATTACTACGGTTATTAATATGCGAACTTATGGGATGGTGTTTGAGCGTATGCCTTTGTTTGT  
CG27Body\_lig-seq 8482 AGCTATTTTTTCTCTTCATTTGGCTGGGGCTTCTTCGATTTTAGGGGCAATTAATTTTATTACTACGGTTATTAATATGCGAACTTATGGGATGGTGTTTGAGCGTATGCCTTTGTTTGT  
CG27Legs\_lig-seq 8459 AGCTATTTTTTCTCTTCATTTGGCTGGGGCTTCTTCGATTTTAGGGGCAATTAATTTTATTACTACGGTTATTAATATGCGAACTTATGGGATGGTGTTTGAGCGTATGCCTTTGTTTGT  
  
CG27Head\_lig-seq 8612 TTGATCTGTAGTGATTACGGCTGTGTTATTATTATTGTCTTTGCCTGTTTTGGCTGGGGCTATTACTATATTGTTAACTGATCGTAATTTTAATACTACTTTTTTTGATCCAGCTGGAGG  
CG27Body\_lig-seq 8602 TTGATCTGTAGTGATTACGGCTGTGTTATTATTATTGTCTTTGCCTGTTTTGGCTGGGGCTATTACTATATTGTTAACTGATCGTAATTTTAATACTACGTTTTTTGATCCGGCTGGAGG  
CG27Legs\_lig-seq 8579 TTGATCTGTAGTGATTACGGCTGTGTTATTATTATTGTCTTTGCCTGTTTTGGCTGGGGCTATTACTATATTGTTAACTGATCGTAATTTTAATACTACGTTTTTTGATCCGGCTGGAGG  
  
CG27Head\_lig-seq 8732 TGGTGATCCTATTTTATATCAGCATTTATTTTGGTTTTTTGGACATCCTGAGGTTTATATTTTAATTCTTCCTGGGTTTGGTATGGTCTCTCATATAATTTCTTATCAGAGAGGAAAGAA  
CG27Body\_lig-seq 8722 TGGTGATCCTATTTTATATCAGCATTTATTTTGGTTTTTTGGACATCCTGAGGTTTATATTTTAATTCTTCCTGGGTTTGGTATGGTCTCTCATATAATTTCTTATCAGAGAGGAAAGAA  
CG27Legs\_lig-seq 8699 TGGTGATCCTATTTTATATCAGCATTTATTTTGGTTTTTTGGACATCCTGAGGTTTATATTTTAATTCTTCCTGGGTTTGGTATGGTCTCTCATATAATTTCTTATCAGAGAGGAAAGAA  
  
CG27Head\_lig-seq 8852 GGAGCCTTTTGGATCTTTAGGAATGATTTATGCTATGGGTGCCATTGGTTTGTTGGGTTTTATTGTTTGGGCTCATCATATGTTTACTGTTGGTATGGATGTCGATACTCGAGCTTATTT  
CG27Body\_lig-seq 8842 GGAGCCTTTTGGATCTTTAGGAATGATTTATGCTATGGGTGCCATTGGTTTGTTGGGTTTTATTGTTTGGGCTCATCATATGTTTACTGTTGGTATGGATGTCGATACTCGAGCTTATTT  
CG27Legs\_lig-seq 8819 GGAGCCTTTTGGATCTTTAGGAATGATTTATGCTATGGGTGCCATTGGTTTGTTGGGTTTTATTGTTTGGGCTCATCATATGTTTACTGTTGGTATGGATGTCGATACTCGAGCTTATTT  
  
CG27Head\_lig-seq 8972 TACTGCTGCTACTATGATTATTGCTGTACCGACAGGTATTAAGATTTTTAGTTGGTTAGCTACTTTGTATGGATCTCGTTTTATTTATGATGTTACTTTGATGTGGTCTTTAGGATTTG-  
CG27Body\_lig-seq 8962 TACTGCTGCTACTATGATTATTGCTGTACCGACAGGTATTAAGATTTTTAGTTGGTTAGCTACTTTGTATGGATCTCGTTTTATTTATGATGTTACTTTGATGTGGTCTTTAGGATTTGT  
CG27Legs\_lig-seq 8939 TACTGCTGCTACTATGATTATTGCTGTACCGACAGGTATTAAGATTTTTAGTTGGTTAGCTACTTTGTATGGATCTCGTTTTATTTATGATGTTACTTTGATGTGGTCTTTAGGATTTGT  
  
CG27Head\_lig-seq 9091 TTTTTTATTTACTGTTGGGGGATTAACCGGGGTAGTTTTGGCTAATTCTTCTATTGATATTATATTGCATGATACTTATTATGTGGTTGCTCATTTTCATTATGTTTTATCAATGGGTGC  
CG27Body\_lig-seq 9082 TTTTTTATTTACTGTTGGGGGATTAACCGGGGTAGTTTTGGCTAATTCTTCTATTGATATTATATTGCATGATACTTATTATGTGGTTGCTCATTTTCATTATGTTTTATCAATGGGTGC  
CG27Legs\_lig-seq 9059 TTTTTTATTTACTGTTGGGGGATTAACCGGGGTAGTTTTGGCTAATTCTTCTATTGATATTATATTGCATGATACTTATTATGTGGTTGCTCATTTTCATTATGTTTTATCAATGGGTGC  
  
CG27Head\_lig-seq 9211 TGTTTTTGCTATTTTGGGGGCAAACACTTTCTGGTTTCCCTTTATTTTTTGGGGTAACTTTTAATACTGCTTTGTTAAAGCTTCATTTTGTTTTGATG-TTTTTGGGGTTAATGTGACTT  
CG27Body\_lig-seq 9202 TGTTTTTGCTATTTTGGGGGCAATCACTTTCTGGTTT-CCCTTATTTTTTGGGGTAACTTTTAATACTGCTTTGTTAAAGCTTCATTTTGTTTTGATGTTTTTTGGGGTTAATGTGACTT  
CG27Legs\_lig-seq 9179 TGTTTTTGCTATTTTGGGGGCAATCACTTTCTGGTTT-CCTTTATTTTTT-GGGTAACTTTTAATACTGCTTTGTTAAAGCTTCATTTTGTTTTGATGTTTTTTGGGGTTAATGTGACTT  
  
CG27Head\_lig-seq 9330 TTTTTCCTCAGCACTTTTTGGGGTTAAGAGGGATACCTCGTCGATATTCTGATTACCCAGATGCTTATGTTGTTTGAAATATGGTGTCTTCTTTTGGTTCTTTAGTTTCTTTATTAGCTA  
CG27Body\_lig-seq 9321 TTTTTCCTCAGCACTTTTTGGGGTTAAGAGGGATACCTCGTCGATATTCTGATTACCCAGATGCTTATGTTGTTTGAAATATGGTGTCTTCTTTTGGTTCTTTAGTTTCTTTATTAGCTA  
CG27Legs\_lig-seq 9297 TTTTTCCTCAGCACTTTTTGGGGTTAAGAGGGATACCTCGTCGATATTCTGATTACCCAGATGCTTATGTTGTTTGAAATATGGTGTCTTCTTTTGGTTCTTTAGTTTCTTTATTAGCTA  
  
CG27Head\_lig-seq 9450 CTTTTTTATATATTTATATTGTTTGGGATGGATTTTTGTGTGAGCGGGATATTTTAACAACTGTAGGTATAACTAGTTCGGTGGAGTGGATTCATTCAACACCCCCTGAAGAGCATACTT  
CG27Body\_lig-seq 9441 CTTTTTTATATATTTATATTGTTTGGGATGGATTTTTGTGTGAGCGGGATATTTTAACAACTGTAGGTATAACTAGTTCGGTGGAGTGGATTCATTCAAC-CCCCCTGAAGAGCATACTT  
CG27Legs\_lig-seq 9417 CTTTTTTATATATTTATATTGTTTGGGATGGATTTTTGTGTGAGCGGGATATTTTAACAACTGTAGGTATAACTAGTTCGGTGGAGTGGATTCATTCAACACCCCCTGAAGAGCATACTT  
  
CG27Head\_lig-seq 9570 TTAATCAGTTGGGGATTTTAATTAGGTAATTTATGGCTACTTGGGGGGGTTTATTATTTCAGGATAGAGTTTCTCCTTTAATGGAACAGTTAATTTTTTTCCACGATCATGCTTTGCTTA  
CG27Body\_lig-seq 9560 TTAATCAGTTGGGGATTTTAATTAGGTAATTTATGGCTACTTGAGGGGGTTTATTATTTCAGGATAGAGTTTCTCCTTTAATGGAACAGTTAATTTTTTTCCACGATCATGCTTTGCTTA  
CG27Legs\_lig-seq 9537 TTAATCAGTTTGGGATTTTAATTAGGTAATTTATGGCTACTTGAGGGGGTTTATTATTTCAGGATAGAGTTTCTCCTTTAATGGAACAGTTAATTTTTTTCCACGATCATGCTTTGCTTA  
  
CG27Head\_lig-seq 9690 TTTTACTTTTAATTACTTCTTTGGTTATTTATATAATTTATATGTTAATAAGAAATATAATATTAAATCGTTTTTTATTGGAGGGTCAGGAGATTGAGATTATTTGGACTGTTTTTCCGG  
CG27Body\_lig-seq 9680 TTTTACTTTTAATTACTTCTTTGGTTATTTATATAATTTATATGTTAATAAGAAATATAATATTAAATCGTTTTTTATTGGAGGGTCAGGAGATTGAGATTATTTGGACTGTTTTTCCGG  
CG27Legs\_lig-seq 9657 TTTTACTTTTAATTACTTCTTTGGTTATTTATATAATTTATATGTTAATAAGAAATATAATATTAAATCGTTTTTTATTGGAGGGTCAGGAGATTGAGATTATTTGGACTGTTTTTCCGG  
  
CG27Head\_lig-seq 9810 CTGTGGTGTTGATTTTTATTGCTTTTCCTTCATTACGTTTGTTATATTTACTTGATGAGGTTAGTTCTCCTGGTTTAACTTTAAAGGTGTTAGGTCATCAATGATATTGAAGTTATGAGT  
CG27Body\_lig-seq 9800 CTGTGGTGTTGATTTTTATTGCTTTTCCTTCATTACGTTTGTTATATTTACTTGATGAGGTTAGTTCTCCTGGTTTAACTTTAAAGGTGTTAGGTCATCAATGATATTGAAGTTATGAGT  
CG27Legs\_lig-seq 9777 CTGTGGTGTTGATTTTTATTGCTTTTCCTTCATTACGTTTGTTATATTTACTTGATGAGGTTAGTTCTCCTGGTTTAACTTTAAAGGTGTTAGGTCATCAATGATATTGAAGTTATGAGT  
  
CG27Head\_lig-seq 9930 ATTCTGATTTTGGGGATTTAGAGTTTGATTCTTATATAAAGATTGATGATGGGTTAGGTGACTTTCGTTTGTTGGATGTTGATAATCGAGTTGTTTTACCAGCAGGTTGTATGGTTCGTT  
CG27Body\_lig-seq 9920 ATTCTGATTTTGGGGATTTAGAGTTTGATTCTTATATAAAGATTGATGATGGGTTAGGTGACTTTCGTTTGTTGGATGTTGATAATCGAGTTGTTTTACCAGCAGGTTGTATGGTTCGTT  
CG27Legs\_lig-seq 9897 ATTCTGATTTTGGGGATTTAGAGTTTGATTCTTATATAAAGATTGATGATGGGTTAGGTGACCTTCGTTTGTTGGATGTTGATAATCGAGTTGTTTTACCAGCAGGTTGTATGGTTCGTT  
  
CG27Head\_lig-seq 10,050 CTTTAATTAGCTCTGTAGATGTGATTCATTCTTGGGCTGTTCCTGGTTTAGGGGTAAAGCTTGATGCTGTTCCCGGGCGATTGAATCAAAGATCTTTTTTTGTTGAGCGGGTTGGTCTTT  
CG27Body\_lig-seq 10,040 CTTTAATTAGCTCTGTAGATGTGATTCATTCTTGGGCTGTTCCTGGTTTAGGGGTAAAGCTTGATGCTGTTCCCGGGCGATTGAATCAAAGATCTTTTTTTGTTGAGCGGGTTGGTC-TT  
CG27Legs\_lig-seq 10,017 CTTTAATTAGCTCTGTAGATGTGATTCATTCTTGGGCTGTTCCTGGTTTAGGGGTAAAGCTTGATGCTGTTCCCGGGCGATTGAATCAAAGATCTTTTTTTGTTGAGCGGGTTGGTC-TT  
  
CG27Head\_lig-seq 10,170 TGGTTTGGTCAGTGTTCTGAGATTTGTGGGGCTAATCATAGTTTTATGCCCGTAGTTATTGAGTCTGTAAGAGGTTCTGGTTTTATAGTTTGATTAGGGGGGCTTATTTACATTGAATGG  
CG27Body\_lig-seq 10,159 TGGTTTGGTCAGTGTTCTGAGATTTGTGGGGCTAATCATAGTTTTATGCCCGTAGTTATTGAGTCTGTAAGAGGTTCTGGTTTTATAGTTTGATTAGGGGGGCTTATTTACATTGAATGG  
CG27Legs\_lig-seq 10,136 TGGTTTGGTCAGTGTTCTGAGATTTGTGGGGCTAATCATAGTTTTATGCCCGTAGTTATTGAGTCTGTAAGAGGTTCTGGTTTTATAGTTTGATTAGGGGGGCTTATTTACATTGAATGG  
  
CG27Head\_lig-seq 10,290 CTGATAAAGGCTGTGGTCTCTTAAATCACATTATAGTATAACTACTTTCAATGGGGCGCTTAGTTAAATTTATAATTTTGATCTGTCAGGTCAGTGTTGCTTTATAGCAGTGCTTAGTGC  
CG27Body\_lig-seq 10,279 CTGATAAAGGCTGTGGTCTCTTAAATCACATTATAGTATAACTACTTTCAATGGGGCGCTTAGTTAAATTTATAATTTTGATCTGTCAGGTCAGTGTTGCTTTATAGCAGTGCTTAGTGC  
CG27Legs\_lig-seq 10,256 CTGATAAAGGCTGTGGTCTCTTAAATCACATTATAGTATAACTACTTTCAATGGGGCGCTTAGTTAAATTTATAATTTTGATCTGTCAGGTCAGTGTTGCTTTATAGCAGTGCTTAGTGC  
  
CG27Head\_lig-seq 10,410 CACAAATATATCCTATTAGATGGATTTTTATTTATTTTTATACTTTATTGGGCTTAATTATGGTTTTAGTTTTGGTGAGATATGTTTATAGTTTTAGGGTAGATAAAATTTTGAGTGAGG  
CG27Body\_lig-seq 10,399 CACAAATATATCCTATTAGATGGATTTTTATTTATTTTTATACTTTATTGGGCTTAATTATGGTTTTAGTTTTGGTGAGATATGTTTATAGTTTTAGGGTAGATAAGATTTTGAGTGAGG  
CG27Legs\_lig-seq 10,376 CACAAATATATCCTATTAGATGGATTTTTATTTATTTTTATACTTTATTGGGCTTAATTATGGTTTTAGTTTTGGTGAGATATGTTTATAGTTTTAGGGTAGATTAGATTTTGAGTGAGG  
  
CG27Head\_lig-seq 10,530 GTG-TTAATGTAGTTGAATTTTTGGTTGTGATAGTTAGTTTATTTTCTGTTTTTGATCCTAGAACTCAGATTTTAAGATTAAATTGATTAAGAGTTTTTCTATGAGTTTTTATTTTTCCT  
CG27Body\_lig-seq 10,519 GTG-TTAATGTAGTTGAA-TTTTGGTTGTGATAGTTAGTTTATTTTCTGTTTTTGATCCTAGAACTCAGATTTTAAGATTAAATTGATTAAGAGTTTTTCTATGAGTTTTTATTTTTCCT  
CG27Legs\_lig-seq 10,496 GTGTTTAATGTAGTTGAA-TTTTGGTTGTGATAGTTAGTTTATTTTCTGTTTTTGATCCTAGAACTCAGATTTTAAGATTAAATTGATTAAGAGTTTTTCTATGAGTTTTTATTTTTCCT  
  
CG27Head\_lig-seq 10,649 TTAGGATATTGGATTATTTCTTCTCGTTATGTTAAGGTATGATTGGTGATTCTATCTAGGTTAATAGAAGAGTTTGTTCTTTTGTTAGGGGGTTCTTCTGGGTATGGGGTAATTTTATTA  
CG27Body\_lig-seq 10,637 TTAGGATATTGGATTATTTCTTCTCGTTATGTTAAGGTATGATTGGTGATTCTATCTAGGTTAATAGAAGAGTTTGTTCTTTTGTTA-GGGGTTCTTCTGGGTATGGGGTAATTTTATTA  
CG27Legs\_lig-seq 10,615 TTAGGATATTGGATTATTTCTTCTCGTTATGTTAAGGTATGATTGGTGATTCTATCTAGGTTAATAGAAGAGTTTGTTCTTTTGTTAGGGGGTTCTTCTGGGTATGGGGTAATTTTATTA  
  
CG27Head\_lig-seq 10,769 GTTCTTGGGTTGTTTAGTTTAATTGTTATAAATAATTTATTTGGTTTAGTTCCTTATGTTTTTACTGGAACTGCTCATTTTGTTATAACTATTAGATTGGCTGTTCCTCTGTGATTGGGT  
CG27Body\_lig-seq 10,756 GTTCTTGGGTTGTTTAGTTTAATTGTTATAAATAATTTATTTGGTTTAGTTCCTTATGTTTTTACTGGAACTGCTCATTTTGTTATAACTATTAGATTGGCTGTTCCTCTGTGATTGGGT  
CG27Legs\_lig-seq 10,735 GTTCTTGGGTTGTTTAGTTTAATTGTTATAAATAATTTATTTGGTTTAGTTCCTTATGTTTTTACTGGAACTGCTCATTTTGTTATAACTATTAGATTGGCTGTTCCTCTGTGATTGGGT  
  
CG27Head\_lig-seq 10,889 TTAATGTTATATGGTTGGATCAATCATACTTTATATATATTTGCACATTTAGTTCCTCAGGGTACACCTGGTTTATTGTTGGTTTTTATGGTTTTAATTGAGAGAATTAGAAACTTAATT  
CG27Body\_lig-seq 10,876 TTAATGTTATATGGTTGGATCAATCATACTTTATATATATTTGCACATTTAGTTCCTCAGGGTACACCTGGTTTATTGTTGGTTTTTATGGTTTTAATTGAGAGAATTAGAAACTTAATT  
CG27Legs\_lig-seq 10,855 TTAATGTTATATGGTTGGATCAATCATACTTTATATATATTTGCACATTTAGTTCCTCAGGGTACACCTGGTTTATTGTTGGTTTTTATGGTTTTAATTGAGAGAATTAGAAACTTAATT  
  
CG27Head\_lig-seq 11,009 CGACCTCTGACTTTATCTGTGCGTTTGGGGGCTAATATAATTGCCGGGCACTTGCTTTTAGTGTTATTAGGGGGTCAGGCTGGGGTTTTAGGTGTAGATGTTATATTGGTTATATTAGGT  
CG27Body\_lig-seq 10,996 CGACCTCTGACTTTATCTGTGCGTTTGGGGGCTAATATAATTGCCGGGCACTTGCTTTTAGTGTTATTAGGGGGTCAAGCTGGGGTTTTAGGTGTAGATGTTATATTGGTTATATTAGGT  
CG27Legs\_lig-seq 10,975 CGACCTCTGACTTTATCTGTGCGTTTGGGGGCTAATATAATTGCCGGGCACTTGCTTTTAGTGTTATTAGGGGGTCAAGCTGGGGTTTTAGGTGTAGATGTTATATTGGTTATATTAGGT  
  
CG27Head\_lig-seq 11,129 CAGATTATATTATTGGTTTTGGAGCTTGCTGTTGCAGTAATTCAGGCTTATGTTTTTGTTACTTTAATAACTTTATATTTTAGAGAGGTAAATTATGATAAGATATACTCATTCATATCA  
CG27Body\_lig-seq 11,116 CAGATTATATTATTGGTTTTGGAGCTTGCTGTTGCAGTAATTCAGGCTTATGTTTTTGTTACTTTAATAACTTTATATTTTAGAGAGGTAAATTATGATAAGATATACTCATTCATATCA  
CG27Legs\_lig-seq 11,095 CAGATTATATTATTGGTTTTGGAGCTTGCTGTTGCAGTAATTCAGGCTTATGTTTTTGTTACTTTAATAACTTTATATTTTAGAGAGGTAAATTATGATAAGATATACTCATTCATATCA  
  
CG27Head\_lig-seq 11,249 TATAGTAGATAAGAGACCTTGACCTGTGTTTGCTGGTTTAGGGGCTCTAAGTTTGACTGTTGGTGGAGTCAATATGTTTTGTGGCGGGGTAGATTTGTTATTCAAGCTTGGTTTGTTAAT  
CG27Body\_lig-seq 11,236 TATAGTAGATAAGAGACCTTGACCTGTGTTTGCTGGTTTAGGGGCTCTAAGTTTGACTGTTGGTGGAGTCAATATGTTTTGTGGCGGGGTAGATTTGTTATTCAAGCTTGGTTTGTTAAT  
CG27Legs\_lig-seq 11,215 TATAGTAGATAAGAGACCTTGACCTGTGTTTGCTGGTTTAGGGGCTCTAAGTTTGACTGTTGGTGGAGTCAATATGTTTTGTGGCGGGGTAGATTTGTTATTCAAGCTTGGTTTGTTAAT  
  
CG27Head\_lig-seq 11,369 TTTGTTTTTAACTGTTTGGCAGTGGTGGCGAGATGTGGTGCGGGAAGGAACTTATTTGGGTTTACATTCTTATTGTGTGGTGCTGGGTTTACGCTGGGGTATAGTTTTATTTATTGTTTC  
CG27Body\_lig-seq 11,356 TTTGTTTTTAACTGTTTGGCAGTGGTGGCGAGATGTGGTGCGGGAAGGAACTTATTTGGGTTTACATTCTTATTGTGTGGTACTGGGTTTACGCTGGGGTATAGTTTTATTTATTGTTTC  
CG27Legs\_lig-seq 11,335 TTTGTTTTTAACTGTTTGGCAGTGGTGGCGAGATGTGGTGCGGGAAGGAACTTATTTGGGTTTACATTCTTATTGTGTGGTACTGGGTTTACGCTGGGGTATAGTTTTATTTATTGTTTC  
  
CG27Head\_lig-seq 11,489 TGAGGTATTTTTTTTTGTTTCTTTTTTTTTGAGCTTTTTTTTCATTCTAGATTATCTCCTGTGTTGGAGATTGGGTCAGTATGACCCCCTGTTGGTATTGAGGTTTTTAATCCTTTTCAG  
CG27Body\_lig-seq 11,476 TGAGGTATTTTTTTTTGTTTC-TTTTTTTTGAGCTTTTTTTTCATTCTAGATTATCTCCTGTGTTGGAGATTGGGTCAGTATGACCCCCTGTTGGTATTGAGGTTTTTAATCCTTTTCAG  
CG27Legs\_lig-seq 11,455 TGAGGTATTTTTTTTTGTTTC-TTTTTTTTGAGC-TTTTTTTCATTCTAGATTATCTCCTGTGTTGGAGATTGGGTCAGTATGACCCCCTGTTGGTATTGAGGTTTTTAATCCTTTTCAG  
  
CG27Head\_lig-seq 11,609 ATTCCACTTTTAAACACTGCTATTTTGTTGGCTTCTGGGGTTAGAGTTACTTGGGCTCATCATTCTTTAATAGAGGGGGATTGAAGTTCTGGGTCTATGGGATTAGCCGTTACTTTTTTA  
CG27Body\_lig-seq 11,595 ATTCCACTTTTAAACACTGCTATTTTGTTGGCTTCTGGGGTTAGAGTTACTTGGGCTCATCATTCTTTAATAG-GGGGGATTGAAGTTCTGGGTCTATGGGATTAGCCGTTACTTTTTTA  
CG27Legs\_lig-seq 11,573 ATTCCACTTTTAAACACTGCTATTTTGTTGGCTTCTGGGGTTAGAGTTACTTGGGCTCATCATTCTTTAATAGAGGGGGATTGAAGTTCTGGGTCTATGGGATTAGCCGTTACTTTTTTA  
  
CG27Head\_lig-seq 11,729 TTAGGTTTTTATTTTTCTGTTCTTCAGGGTTATGAGTATTATGAGGCTAGTTTTAGAATTGCTGATTCAGTTTATGGTTCTGTTTTTTTTATAGCAACTGGGTTTCATGGTCTTCATGTT  
CG27Body\_lig-seq 11,714 TTAGGTTTTTATTTTTCTGTTCTTCAGGGTTATGAGTATTATGAGGCTAGTTTTAGAATTGCTGATTCAGTTTATGGTTCTGTTTTTTTTATAGCAACTGGGTTTCATGGTCTTCATGTT  
CG27Legs\_lig-seq 11,693 TTAGGTTTTTATTTTTCTGTTCTTCAGGGTTATGAGTATTATGAGGCTAGTTTTAGAATTGCTGATTCAGTTTATGGTTCTGTTTTTTTTATAGCAACTGGGTTTCATGGTCTTCATGTT  
  
CG27Head\_lig-seq 11,849 TTAATTGGTTCTAGATTTTTGTTGGTTTGTTTAGTTCGCCAGGTATTAAGACATTATTCTGAGACTCATCATTTTGGTTTTGAGGCTGCGGCTTGGTATTGGCATTTTGTTGATGTAGTT  
CG27Body\_lig-seq 11,834 TTAATTGGTTCTAGATTTTTGTTGGTTTGTTTAGTTCGCCAGGTATTAAGACATTATTCTGAGACTCATCATTTTGGTTTTGAGGCTGCGGCTTGGTATTGGCATTTTGTTGATGTAGTT  
CG27Legs\_lig-seq 11,813 TTAATTGGTTCTAGATTTTTGTTGGTTTGTTTAGTTCGCCAGGTATTAAGACATTATTCTGAGACTCATCATTTTGGTTTTGAGGCTGCGGCTTGGTATTGGCATTTTGTTGATGTAGTT  
  
CG27Head\_lig-seq 11,969 TGATTATTTTTATATGTATCTATTTACTGATGAGGGGGTTAAGTTTGTTGAGTATGATAAGTATAGCTAATTTCCAATTAGATGGTTTGAGAATTTGAAACAAATAATGGG-CGTTTTAG  
CG27Body\_lig-seq 11,954 TGATTATTTTTATATGTATCTATTTACTGATGAGGGGGTTAAGTTTGTTGAGTATGATAAGTATAGCTAATTTCCAATTAGATGGTTTGAGAATTTGAAACAAATAATGGGCCGTTTTAG  
CG27Legs\_lig-seq 11,933 TGATTATTTTTATATGTATCTATTTACTGATGAGGGGGTTAAGTTTGTTGAGTATGATAAGTATAGCTAATTTCCAATTAGATGGTTTGAGAATTTGAAACAAATAATGGG-CGTTTTAG  
  
CG27Head\_lig-seq 12,088 TAATTTATTCTATTTTTTTTTTGGTTGGGCTTGTTCTGTCCTTAGTGGCTTGGCGTATTGTTTCGTTAGGTTTGAGAAGAGAGGATAAATTATCTTCGTTTGAGTGTGGTTTTGTTTCCT  
CG27Body\_lig-seq 12,074 TAATTTATTCTATTTTTTTTTTGGTTGGGCTTGTTCTGTCCTTAGTGGCTTGGCGTATTGTTTCGTTAGGTTTGAGAAGAGAGGATAAATTATCTTCGTTTGAGTGTGGTTTTGTCTCCT  
CG27Legs\_lig-seq 12,052 TAATTTATTCTA-TTTTTTTTTGGTTGGGCTTGTTCTGTCCTTAGTGGCTTGGCGTATTGTTTCTGTTAGGTTTAGAAGAGAGGATAAATTATCTTCGTTTGAGTGTGGTTTTGTCTCCT  
  
CG27Head\_lig-seq 12,208 TTCAAGGTTCTCGGTTAGCTTTTTCTTTACAGTTTTTTTTAATTGCTATTGTTTTTCTGATTTTTGATGTTGAGATTGCTTTAGTTTTACCAATTCCGGTAATTTTTGAGTTAGTGAGTT  
CG27Body\_lig-seq 12,194 TTCAAGGTTCTCGGTTAGCTTTTTCTTTACAGTTTTTTTTAATTGCTATTGTTTTTCTGATTTTTGATGTTGAGATTGCTTTAGTTTTACCAATTCCGGTAATTTTTGAGTTAGTGAGTT  
CG27Legs\_lig-seq 12,171 TTCAAGGTTCTCGGTTAGCTTTTTCTTTACAGTTTTTTTTAATTGCTATTGTTTTTCTGATTTTTGATGTTGAGATTGCTTTAGTTTTACCAATTCCTGTAATTTTTGAGTTAGTGAGTT  
  
CG27Head\_lig-seq 12,328 ATGAGGTCGTTTTTTTAGTTTTTTCAGTTTTTGGATTTGTTGTGTTGGGGGGTTTGTATTATGAGTGAGGTTGTGGGGTGTTGGATTGAGCATATTAGGTTTGTATTTTAAATTAAAATT  
CG27Body\_lig-seq 12,314 ATGAGGTGGTTTTTTTAGTTTTTTCAGTTTTTGGATTTGTTGTGTTGGGGGGTTTGTATTATGAGTGAGGTTGTGGGGTGTTGGATTGAGCATATTAGGTTTGTATTTTAAATTAAAATT  
CG27Legs\_lig-seq 12,291 ATGAGGTGGTTTTTTTAGTTTTTTCAGTTTTTGGATTTGTTGTGTT-GGGGGTTTGTATTATGAGTGAGGTTGTGGGGTGTTGGATTGAGCATATTAGGTTTGTATTTTAAATTAAAATT  
  
CG27Head\_lig-seq 12,448 TTTAGTTTGCAATTAATTGATGCATTATGCCAAGCCTGTAAGTGAGAAGCTATTATGCGATCAGTTTCGGCCTGATTATTTTGGACTATTTGTTCCTTACTTAGTAAAGGCCGAATGGCG  
CG27Body\_lig-seq 12,434 TTTAGTTTGCAATTAATTGATGCATTATGCCAAGCCTGTAAGTGAGAAGCTATTATGCGATCAGTTTCGGCCTGATTATTTTGGACTATTTGTTCCTTACTTAGTAAAGGCCGAATGGCG  
CG27Legs\_lig-seq 12,410 TTTAGTTTGCAATTAATTGATGCATTATGCCAAGCCTGTAAGTGAGAAGCTATTATGCGATCAGTTTCGGCCTGATTATTTTGGACTATTTGTTCCTTACTTAGTAAAGGCCGAATGGCG  
  
CG27Head\_lig-seq 12,568 TTTTTTTGTTAAGAATATGGTTGGGATTAAATATCTCTTACTAAGGATTAATTTAAAATATAAGCCGCTAACTTATAAAGAGCCCTTGATTGGGTTAATAGTCTCTTTTTATGGTGTAAT  
CG27Body\_lig-seq 12,554 TTTTTTTGTTAAGAATATGGTTGGGATTAAATATCTCTTACTAAGGATTAATTTAAAATATAAGCCGCTAACTTATAAAGAGCCCTTGATTGGGTTAATAGTCTCTTTTTATGGTGTAAT  
CG27Legs\_lig-seq 12,530 TTTTTTTGTTAAGAATATGGTTGGGATTAAATATCTCTTACTAAGGATTAATTTAAAATATAAGCCGCTAACTTATAAAGAGCCCTTGATTGGGTTAATAGTCTCTTTTTATGGTGTAAT  
  
CG27Head\_lig-seq 12,688 TATAACACGTAACTTTTTCGTGGTTAAGAAAGTTTTGCTTAAAAAGTTTGGTTTTTAGCTTATTGTTGTTTTTTTTCTATTGTTATTAGAGTGATTTTTTTCTCAGGTTGTGAACCCTAT  
CG27Body\_lig-seq 12,674 TATAACACGTAACTTTTTCGTGGTTAAGAAAGTTTTGCTTAAAAAGTTTGGTTTTTAGCTTATTGTTG-TTTTTTTCTATTGTTATTAGAGTGA-TTTTTTCTCAGGTTGTGAACCCTAT  
CG27Legs\_lig-seq 12,650 TATAACACGTAACTTTTTCGTGGTTAAGAAAGTTTTGCTTAAAAAGTTTGGTTTTTAGCTTATTGTTG-TTTTTTTCTATTGTTATTAGAGTGA-TTTTTTCTCAGGTTGTGAACCCTAT  
  
CG27Head\_lig-seq 12,808 ATTATTGGCTTATTTGGTGGTTGGGGGGGGGGTTGTGGTGATAATTTCTTTAGGGTTGATTTTTTCTACTTTCTGATTGAGATATATTGTATTGTTGACTTTTTTTAGGGGGGGTTGTTG  
CG27Body\_lig-seq 12,792 ATTATTGGCTTATTTGGTGG-TGGGGTGGGGGTTGTGGTGATAATTTCTTTAGGGTTGATTTTTTCTACTTTCTGATTGAGATATATTGTATTGTTGACTTTTTTA---GGGGGTTGTTG  
CG27Legs\_lig-seq 12,768 ATTATTGGCTTATTTGGTGGTTGGCGGGGGGGTTGTGGTGATAATTTCTTTAGGGTTGATTTTTTCTACTTTCTGATTGAGATATATTGTATTGTTGACTTTTTTA--GGGGGGTTGTTG  
  
CG27Head\_lig-seq 12,928 GTATTATTTGTTTATGTTGCTTCTTTATCACCAAATGAGCCGGTGTTGGGGGGTTGGAGGTGGA-TTGGTTGTGGTAAGAGGATTAGTTTTATTTTTTAGTTTTAAATTGGGAGTCTACT  
CG27Body\_lig-seq 12,908 GTATTATTTGTTTATGTTGCTTCTTTATCACCAAATGAGCCGGTGTT-GGGGGTTGGAGGTGGAGTTGGTTGTGGTAAGAGGATTAGTTTTA-TTTTTAGTTTTAAATTGGGAGTCTACT  
CG27Legs\_lig-seq 12,886 GTATTATTTGTTTATGTTGCTTCTTTATCACCAAATGAGCCGGTGTT-GGGGGTTGGAGGTGGA-TTGGTTGTGGTAAGAGGATTAGTTTTA-TTTTTAGTTTTAAATTGGGAGTCTACT  
  
CG27Head\_lig-seq 13,047 TCTGATTTAGGTTTTAGAGCTGGTTTTTTGTTTTTTAATAAAGATGTACTTAGGGAATATAGAGGGTTTTATAGTTTTACTTATTTTATATTTATTTTTGGTTCTATTAGTATCTGTTGA  
CG27Body\_lig-seq 13,026 TCTGATTTAGGTTTTAGAGCTGGTTTTTTG-TTTTTAATAAAGATGTACTTAGGGAATATAGAGGGTTTTATAGTTTTACTTATTTTATATTTATTTTTGGTTCTATTAGTATCTGTTGA  
CG27Legs\_lig-seq 13,003 TCTGATTTAGGTTTTAGAGCTGGTTTTTTG-TTTTTAATAAAGATGTACTTAGGGAATATAGAGGGTTTTATAGTTTTACTTATTTTATATTTATTTTTGGTTCTATTAGTATCTGTTGA  
  
CG27Head\_lig-seq 13,167 TTTGAGTTTAATTGTTATTGGGCCACTAAGGGTACTATATGATAAGGGGAAGGGAAGGAAAATTTATTAGTTAGAGGTTTTATAGCTTTCTTAGTTGAGTTACCAACACCTAGAAGAATT  
CG27Body\_lig-seq 13,145 TTTGAGTTTAATTGTTATTGGGCCACTAAGGGTACTATATGATAAGGGGAAGGGAAGGAAAATTTATTAGTTAGAGGTTTTATAGCTTTCTTAGTTGAGTTACCAACACCTAGAAGAATT  
CG27Legs\_lig-seq 13,122 TTTGAGTTTAATTGTTATTGGGCCACTAAGGGTACTATATGATAAGGGGA--GGAAGGAAAATTTATTAGTTAGAGGTTTTATAGCTTTCTTAGTTGAGTTACCAACACCTAGAAGAATT  
  
CG27Head\_lig-seq 13,287 AGTTATATGTGGAATTTTGGTTCTATATTAGCATTTTGTTTAGGTTTGCAGATTTTAACTGGTCTTTTTTTGGCTATGCATTATAGAGCTCATGTGGACACAGCTTTTTTTAGTGTTGTT  
CG27Body\_lig-seq 13,265 AGTTATATGTGGAATTTTGGTTCTATATTAGCATTTTGTTTAGGTTTGCAGATTTTAACTGGTC-TTTTTTGGCTATGCATTATAGAGCTCATGTGGACACAGCTTTTTTTAGTGTTGTT  
CG27Legs\_lig-seq 13,240 AGTTATATGTGGAATTTTGGTTCTATATTAGCATTTTGTTTAGGTTTGCAGATTTTAACTGGTCTTTTTTTGGCTATGCATTATAGAGCTCATGTGGACACAGCTTTTTTTAGTGTTGTT  
  
CG27Head\_lig-seq 13,407 CATTTGTCTCGTGATGTTAATTATGGGTGGTTGTTGCATTTTATTCATGCCAACGGGGCTAGATTGTTTTTTGTTTGTTTGTATATGCATGTTGGTCGGGGTTTTATATTATGGTTCTTA  
CG27Body\_lig-seq 13,384 CATTTGTCTCGTGATGTTAATTATGGGTGGTTGTTGCATTTTATTCATGCCAACGGGGCTAGATTGTTTTTTGTTTGTTTGTATATGCATGTTGGTCGGGG-TTTATATTATGGTTCTTA  
CG27Legs\_lig-seq 13,360 CATTTGTCTCGTGATGTTAATTATGGGTGGTTGTTGCATTTTATTCATGCCAACGGGGCTAGATTGTTTTTTGTTTGTTTGTATATGCATGTTGGTCGGGG-TTTTTATTATGGTTCTTA  
  
CG27Head\_lig-seq 13,527 TGTTTTATTTCATGTATGAAGCTCTGGGATTAGTATTCTATTAATTACTATATTAACTGCTTTTTTTTAGGTTATGTATTACCATGGGGACAGATGTCTTTCTGGGGGGCCACGGTAATT  
CG27Body\_lig-seq 13,503 TGTTTTATTTCATGTATGAAGCTCTGGGATTAGTATTCTATTAATTACTATATTAACTGC--TTTTTTAGGTTATGTATTACCATGGGGACAGATGTCTTTCTGGGGGGCCACGGTAATT  
CG27Legs\_lig-seq 13,479 TGTTTTATTTCATGTATGAAGCTCTGGGATTAGTATTCTATTAATTACTATATTAACTGC--TTTTTTAGGTTATGTATTACCATGGGGACAGATGTCTTTCTGGGGGGCCACGGTAATT  
  
CG27Head\_lig-seq 13,647 ACTAATTTATTATCTGCTGTTCCATATTGGGGGGTGGACCTAGTACAGTGGGTATGGGGGGGTTTTTCTGTTGGAAATCCTACTTTAGTTCGTTTTTTTGCTTTTCATTTCTTATTTCCT  
CG27Body\_lig-seq 13,621 ACTAATTTATTATCTGCTGTTCCATATTGGGGGGTGGACCTAGTACAGTGGGTATGGGGGGGTTTTTCTGTTGGAAATCCTACTTTAGTTCGTTTTTTTGCTTTTCATTTCTTATTTCCT  
CG27Legs\_lig-seq 13,597 ACTAATTTATTATCTGCTGTTCCATATTTGGGGGTGGACCTAGTACAGTGGGTATGGGGGGGTTTTTCTGTTGGAAATCCTACTTTAGTTCGTTTTTTTGCTTTTCATTTCTTATTTCCT  
  
CG27Head\_lig-seq 13,767 TTTTTAATTGTGGGGTTAGTGTTGGTTCACTTGCTTTTTTTACATGAGACTGGCTCTAGTATGCCTCTAGGTTTAAATTCTGATGTATTAAAGATTGAATTTCATCCTTATTTTTAGAGT  
CG27Body\_lig-seq 13,741 TTTTTAATTGTGGGGTTAGTGTTGGTTCACTTGCTCTTTTTACATGAGACTGGCTCTAGTATGCCTCTAGGTTTAAATTCTGATGTATTAAAGATTGAATTTCATCCTTA-TTTTAGAGT  
CG27Legs\_lig-seq 13,717 TTTTTAATTGTGGGGTTAGTGTTGGTTCACTTGCTTTTTTTACATGAGACTGGCTCTAGTATGCCTCTAGGTTTAAATTCTGATGTATTAAAGATTGAATTTCATCCTTA-TTTTAGAGT  
  
CG27Head\_lig-seq 13,887 AAGGGATCTATATGGTTTATGTTGGGTGTTTGGTGGTTTGTTATTTATTGTTTTATTTTATCCTAATGTTTTAGGGGACTCTGAGAATTTTATTTCGGCTAATCCTTTGGTTACTCCAGA  
CG27Body\_lig-seq 13,860 AAGGGATCTATATGGTTTATGTTGGGTGTTTGGTGGTTTGTTATTTATTGTTTTATTTTATCCTAATGTTTTAGGGGACTCTGAGAATTTTATTTCGGCTAATCCTTTGGTTACTCCAGA  
CG27Legs\_lig-seq 13,836 AAGGGATCTATATGGTTTATGTTGGGTGTTTGGTGGTTTGTTATTTATTGTTTTATTTTATCCTAATGTTTTAGGGGACTCTGAGAATTTTATTTCGGCTAATCCTTTTGTTACTCCAGA  
  
CG27Head\_lig-seq 14,007 GCACATTCAACCTGAGTGGTATTTTTTGTTTGCTTATGCTATTTTACGTTCTATTCCGAATAAGCTTGGTGGTGTGGTGGCTTTAGTTATGTCTATTTTGATTTTATTTATTTGCTCGTT  
CG27Body\_lig-seq 13,980 GCACATTCAACCTGAGTGGTATTTTTTGTTTGCTTATGCTATTTTACGTTCTATTCCGAATAAGCTTGGTGGTGTGGTGGCTTTAGTTATGTCTATTTTGATTTTATTTATTTGCTCGTT  
CG27Legs\_lig-seq 13,956 GCACATTCAACCTGAGTGGTATTTTTTGTTTGCTTATGCTATTTTACGTTCTATTCCGAATAAGCTTGGTGGTGTGGTGGCTTTAGTTATGTCTATTTTGATTTTATTTATTTGCTCGTT  
  
CG27Head\_lig-seq 14,127 ATTTACTGGCGAGCTAAAGTTGGGGGGTTGGTTTATTTTTTGGCGGGTTATGTTTTGATGTTTTGTAGTTATTTTTATTTTATTGACTTGGATTGGGGCTCGTCCTGTGGAGGATCCTTA  
CG27Body\_lig-seq 14,100 ATTTACTGGCGAGCTAAAGTTGGGGGGTTGGTTTATTTTTTGGCGGGTTATGTTTTGATGTTTTGTAGTTATTTTTATTTTATTGACTTGGATTGGGGCTCGTCCTGTGGAGGATCCTTA  
CG27Legs\_lig-seq 14,076 ATTTACTGGCGAGCTAAAGTT-GGGGGTTGGTTTATTTTTTGGCGGGTTATGTTTTGATGTTTTGTAGTTATTTTTATTTTATTGACTTGGATTGGGGCTCGTCCTGTGGAGGATCCTTA  
  
CG27Head\_lig-seq 14,247 TGTTTTGATTGGGCAAAACTTAACTGTGTTGTATTTTTTAAATTTTGTCTTAATGGGGTTTATCCCATAAGATTGGTTAATTACCATCTTGATTGAGTATTTTGAAAATACTATTTAAAA  
CG27Body\_lig-seq 14,220 TGTTTTGATTGGGCAAAACTTAACTGTGTTGTATTTCTTAAATTTTGTCTTAAT-GGGTTTATCCCATAAGATTGGTTAATTACCATCTTGATTGAGTATTTTGAAAATACTATTTAAAA  
CG27Legs\_lig-seq 14,195 TGTTTTGATTGGGCAAAACTTAACTGTGTTGTATTTCTTAAATTTTGTCTTAAT-GGGTTTATCCCATAAGATTGGTTAATTACCATCTTGATTGAGTATTTTGAAAATACTATTTAAAA  
  
CG27Head\_lig-seq 14,367 ACTAATGTTTTTTAGTTGGTTGAGCCGGTCTAGTTTAAGAAAAATATTAATTTTGTAAATTAATGATCCTGTGTGGATTGGTAAG-------ATTTGGGATATCCCTTGGGTAAAACC--  
CG27Body\_lig-seq 14,339 ACTAATG-TTTTTAGTTGGTTGAGCCGGTCTAGTTTAAGAAAAATATTAATTTTGTAAATTAATGATCCTGTGTGGATTGGTAAG-------ATTTGGGCTT--------AT---CCCGC  
CG27Legs\_lig-seq 14,314 ACTAATG-TTTTTAGTTGGTTGAGCCGGTCTAGTTTAAGAAAAATATTAATTTTGTAAATTAATGATCCTGTGTGGATTGGTGGAGTAAGATGTTTGGGTTA--------ATAAACCC--  
  
CG27Head\_lig-seq 14,477 ---CCTTAGTAAAATCATTAATTAAAGGCCTGCTTTACTAAGGAGGGCC-TAAAATAAGATAGTGTTAGTAATAAGAGAAATAAAAAACATTAGGTAGAAGCATACATGTGGAGTGAAGA  
CG27Body\_lig-seq 14,440 GTACCCTAGTAAAATCATTAATTAAAGGCCT--TTTACTAATAAGGGCT-TAAAATAAGATAGTGTTAGTAATAAGAGAAATAAAAAACATTAGGTAGAAGCATACATGTGGAGTGAAGA  
CG27Legs\_lig-seq 14,422 ---CCTTAGTAAAATCATTAATTAAAGGCCTTCTTTACTAATGAGGGCTCTAAAATAAGATAGTGTTAGTAATAAGAGAAATAAAAAACATTAGGTAGAAGCATACATGTGGAGTGAAGA  
  
CG27Head\_lig-seq 14,594 ACAACTTTATGAGATCACTATAAAAATAACAATTCAGGTGTTGGGAGAAAGGATGGACCTATGGTTACTTAGGAGTAGTATTTTAGACCTCTGGCCTATCGTCAATAGTACACCCTAGAA  
CG27Body\_lig-seq 14,557 ACAACTTTATGAGATCACTATAAAAATAACAATTCAGGTGTTGGGAGAAAGGATGGACCTATGGTTACTTAGGAGTAGTATTTTAGACCTCTGGCCTATCGTCAATAGTACACCCTAGAA  
CG27Legs\_lig-seq 14,540 ACAACTTTATGAGATCACTATAAAAATAACAATTCAGGTGTTGGGA-AAAGGATGGACCTATGGTTACTTAGGAGTAGTATTTTAGACCTCTGGCCTATCGTCAATAGTACACCCTAGAA  
  
CG27Head\_lig-seq 14,714 TGAATAATAAAA--CTGTCTTGTGGGTTAGAGCCTCTATCAGCTCATCTGTCGTTATAAGCCTATACGTAATAATAATATAATAACAATAATATCTTATA----GAGGCGGGGGAGGGAT  
CG27Body\_lig-seq 14,677 TGAATAATAAAAACCTGTCTTGTGGGTTAGAGCT-CTATCAGCTCATCTGTCGTTATAAGCCTATACGTAATAATAATATAATAACAATAATATCTTATAGTGGGCGGGGGGGAGGGGTT  
CG27Legs\_lig-seq 14,659 TGAATAATAAAAACCTGTCTTGTGGGTTAGAGCTCCTATCAGCTCATCTGTCGTTATAAGCCTATACGTAATAATAATATAATAACAATAATATCTTATA----GAGGCGGGGGAGGGTT  
  
CG27Head\_lig-seq 14,828 ATAGTGGAGATTTGGGGGTTAATATAATATTGCTAAGTATCGGTTTACCCCCAGGAAGAGACCTCTTAATGCCAGCTACTACTTTGTAAATTGTCGGGGCCGTTTGTACGGGCGGGAGAG  
CG27Body\_lig-seq 14,796 AATTTTTGAGGTTGGGGGTTAATAAATTTATTTTAAGTATTGGTTTACCCCCAGGAAGAGACCTCTTAATGCCAGCTACTACTTTGTAAATTGTCGGGGCCGTTTGTACGGGCGGGAGAG  
CG27Legs\_lig-seq 14,775 AATTTT--GAGTTGGGGGTTAATAAAATTTATTTAAGTATCGGTTTACCCCCAGGAAGAGACCTCTTAATGCCAGCTACTACTTTGTAAATTGTCGGGGCCGTTTGTACGGGCGGGAGAG  
  
CG27Head\_lig-seq 14,948 CTGGGGGGTTGAGCGCGAGGGGGGTCAGGTTTTATGTGTGTTTATGTGTTGTGGTTTATTAGGGTGGGCCAACTTAGGTCGGATATAGCTTGGGTGGATGGGTGGGGTAATGATATGTGC  
CG27Body\_lig-seq 14,916 CTGGGGGGTTGAGCGCGAGGGGGGTCAGGTTTTATGTGTGTTTATGTGTTGTGGTTTATTAGGGTGGGCCAACTTAGGTCGGATATAGCTTGGGTGGATGGGTGGGGTAATGATATGTGC  
CG27Legs\_lig-seq 14,893 CTGGGGGGTTGAGCGCGAGGGGGGTCAGGTTTTATGTGTGTTTATGTGTTGTGGTTTATTAGGGTGGGCCAACTTAGGTCGGATATAGCTTGGGTGGATGGGTGGGGTAATGATATGTGC  
  
CG27Head\_lig-seq 15,068 GAGCGGGTATATATGCCTACTAGCTGGTGTGGGGGTTTATCCTTTATTTGGTGGTTGGGTTTGTTTTAGTGTGTTTTCTTAGTCTTGTTATTTTTAAGTTGAGCTTTTACTTATAAATGT  
CG27Body\_lig-seq 15,036 GAGCGGGTATATATGCCTACTAGCTGGTGTGGGGGTTTATCCTTTATTTGGTGGTTGGGTTTGTTTTAGTGTGTTTTCTTAGTCTTGTTATTTTTAAGTTGAGCTTTTACTTATAAATGT  
CG27Legs\_lig-seq 15,013 GAGCGGGTATATATGCCTACTAGCTGGTGTGGGGGTTTATCCTTTATTTGGTGGTTGGGTTTGTTTTAGTGTGTTTTCTTAGTCTTGTTATTTTTAAGTTGAGCTTTTACTTATAAATGT  
  
CG27Head\_lig-seq 15,188 GATACTAATAGGGGGAAGTATTATTTTCCTAAGTTTGGGTTTGCCTCATCTTGTTCTGGAGAAAAAAAATACCAAGAGTAAAACATCAAATTATAGGCATGTAAGAGTTTTAGTTTCAGA  
CG27Body\_lig-seq 15,156 GATACTAATAGGGGGAAGTATTATTTTCCTAAGTTTGGGTTTGCCTCATCTTGTTCTGGAGAAAAAAAATACCAAGAGTAAAACATCAAATTATAGGCATGTAAGAGTTTTAGTTTCAGA  
CG27Legs\_lig-seq 15,133 GATACTAATAGGGGGAAGTATTATTTTCCTAAGTTTGGGTTTGCCTCATCTTGTTCTGGAGAAAAAAAATACCAAGAGTAAAACATCAAATTATAGGCATGTAAGAGTTTTAGTTTCAGA  
  
CG27Head\_lig-seq 15,308 ACAAAACCCGGATGGTTTTTTGT  
CG27Body\_lig-seq 15,276 ACAAAACCCGGATGGTTTTTTGT  
CG27Legs\_lig-seq 15,253 ACAAAACCCGGATGGTTTTTTGT  
  
